# Targeting lysine-specific demethylase 1 inhibits melanoma metastasis via the *NF2*-Hippo-YAP pathway

**DOI:** 10.1038/s41419-026-08872-1

**Published:** 2026-05-16

**Authors:** Han Yang, Yanli Ding, Yuxing Wang, Shuzhen Wei, Weizhen Cao, Yichao Xu, Pengxing He, Linlin Chang

**Affiliations:** 1 The Affiliated Cancer Hospital of Zhengzhou University & Henan Cancer Hospital, Zhengzhou, China; 2 School of Pharmaceutical Sciences, Zhengzhou University, Zhengzhou, China; 3 Henan Provincial Key Laboratory of Anticancer Drug Research, Engineering Research Center for Tumour Precision Medicine and Comprehensive Evaluation, Henan Cancer Hospital, Zhengzhou, China

**Keywords:** Melanoma, Epithelial-mesenchymal transition

## Abstract

Melanoma, originating from the malignant change in skin melanocytes, is highly metastatic, yet effective treatments to prevent its spread are still lacking. Lysine-specific histone demethylase 1 (LSD1) functions as an epigenetic modifier; however, its role in melanoma remains incompletely elucidated. In this study, we determined that LSD1 expression is upregulated in metastatic melanoma relative to primary melanoma, which is indicative of a poor prognosis. Subsequent experiments revealed that targeting LSD1 effectively suppresses melanoma metastasis in both in vitro and in vivo models. Mechanistically, the pharmacological or genetic inhibition of LSD1 induces the phosphorylation and subsequent degradation of Yes-associated protein (YAP), a critical component of the Hippo signaling pathway that is strongly linked to tumor metastasis. Furthermore, ChIP-qPCR analysis indicates that the LSD1 inhibition enhances H3K4me2 modification at the promoter regions of *NF2* and *LATS1/2*, thereby promoting their transcriptional activation. This results in increased expression of NF2 (encoded by *NF2*) and LATS1/2, ultimately activating the Hippo pathway. These findings not only enhance our understanding of the molecular mechanisms driving melanoma metastasis but also establish a novel theoretical basis for the development of LSD1 inhibitors and targeted therapeutic strategies for melanoma.

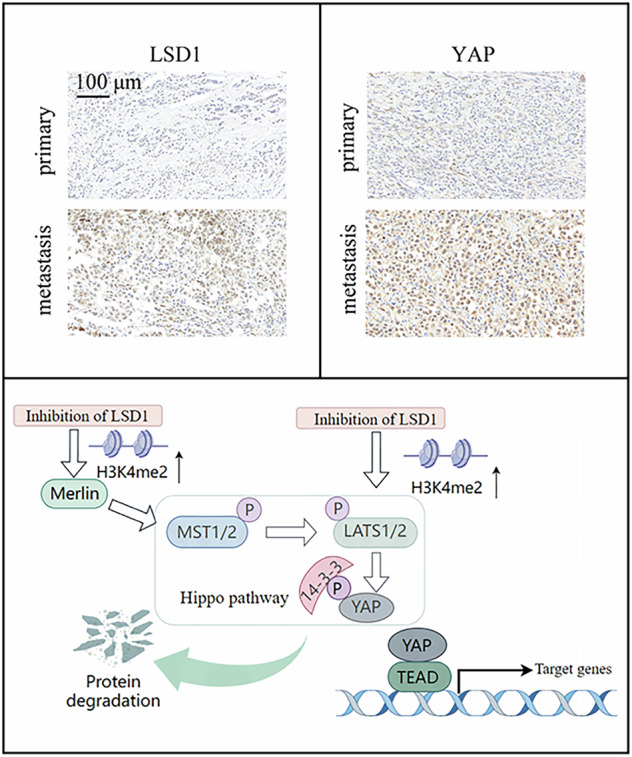

## Introduction

Melanoma, the most lethal form of skin cancer, is characterized by a high potential for invasion and metastatic spread [1]. The malignant transformation of melanocytes involves complex genetic and protein mutations that confer increased proliferative and invasive potential, ultimately resulting in tumor dissemination and metastasis [2]. Although melanoma is not an epithelial cancer, melanoma cells undergo a phenotypic switch similar to epithelial-mesenchymal transition (EMT) observed in epithelial tumors, contributing to tumor progression [3, 4]. A significant limitation of current melanoma treatments is the inability of these treatments to effectively inhibit metastasis. Therefore, identifying molecular targets and developing drugs to inhibit melanoma metastasis is crucial for improving treatment outcomes.

In 2004, Lysine-Specific Histone Demethylase 1A (LSD1, KDM1A) was discovered as the first histone demethylase [5]. LSD1 utilizes FAD as a cofactor to specifically remove methyl groups from lysine (or arginine), thereby modulating gene transcriptional activity. An increasing number of studies have shown that LSD1 plays a pivotal role in a wide range of cellular processes, such as cell proliferation [6], EMT [7], chromosome segregation [8], metabolism [9], stem cell pluripotency regulation [10], and embryonic development [11]. LSD1 dysregulation is closely associated with human cancer development [12]. Knocking out or inhibiting LSD1 can effectively inhibit tumor development and significantly reduce the migration and invasion of breast cancer [13], prostate cancer [14], lung cancer [15, 16], ovarian cancer [17], colon cancer [18], glioblastoma [19], and gastric cancer [20]. In this study, we found that LSD1 exhibits the highest expression levels among common methylation modification enzymes in primary melanoma patients and further upregulates in metastatic melanoma patients. These findings highlight LSD1 as a potential therapeutic target for preventing EMT and tumor metastasis in melanoma.

YAP is the main effector downstream of the Hippo signaling pathway, persistent overactivation of which in a variety of solid tumors can induce tumor cell stemness, proliferation, drug resistance, and metastasis [21–23]. Upon receiving physiological or non-physiological stress signals, upstream membrane protein receptors (e.g., LATS1/2) of the Hippo signaling pathway activate the signaling cascade. This leads to the phosphorylation of downstream transcriptional coactivators, Yes-associated protein (YAP) and transcriptional coactivator with PDZ-binding motif (TAZ). Phosphorylated YAP and TAZ are then bound by the cytoskeletal protein 14-3-3, sequestering them in the cytoplasm and preventing nuclear translocation, which inhibits their interaction with other transcription factors, especially the nuclear DNA-binding partner TEAD (transcriptional enhancement associated domains), thereby limiting the regulation of downstream target genes. Despite the therapeutic potential of transcription factor targeting, it is hard to directly target transcription factors because of the complex binding modes of nucleic acid-protein interactions. Therefore, elucidating the upstream molecular mechanisms of the Hippo-YAP signaling pathway may pave the way for novel therapies against metastatic melanoma.

The objective of this study is to investigate the effects of LSD1 on melanoma cell metastasis, with a particular focus on the Hippo signaling pathway. We showed that inhibiting LSD1 suppresses melanoma metastasis both in vitro and in vivo through the NF2-Hippo-YAP signaling pathway. This process involves the targeting of LSD1-mediated transcriptional activation of NF2 and LATS1/2, which stabilizes the Hippo signaling pathway. Consequently, YAP undergoes phosphorylation and degradation, reducing its nuclear translocation and thereby preventing oncogenic signaling and melanoma metastasis.

## Materials and methods

### Cell culture

Melanoma cell line B16 was purchased from the Cell Bank of the Chinese Academy of Sciences, and A875 cell line was purchased from the Institute of Basic Medicine, Chinese Academy of Medical Sciences. A375, MEL-2, MEL-28, and B16-F10 cells were purchased from Ubigene Biosciences (Guangzhou, China). All of the cells were cultured in DMEM medium containing 10% FBS in a 37 °C incubator with 5% CO_2_. All these cell lines have undergone STR molecular analysis and mycoplasma contamination testing.

### siRNA and plasmid transfection

The cells were seeded in 6-well plates and transfected after the cell density reached 50–60%. LSD1, NF2, and YAP siRNAs were purchased from GenePharma (Shanghai, China), and LATS1/2 siRNAs were purchased from Tsingke (Beijing, China). The transfection reagent Entranster TM-H4000 was purchased from Engreen (Beijing, China). YAP overexpression plasmid Pcmv-Yap1 (mouse)-3×FLAG-Neo was purchased from Miaoling Biology (Wuhan, China). After transfection, resistance screening was performed with G418 at a concentration of 800 μg/mL. The siRNA sequences are shown in Table S1.

### Recombinant plasmid construction and lentivirus packaging

The NF2 sgRNA sequence was inserted into the Lenti CRISPR v2 plasmid (Cat#52961, Addgene) to construct a recombinant plasmid, which was then packaged into lentivirus. The system comprised pCMV-VSV-G (Cat#8454, Addgene) and psPAX2 (Cat#12260, Addgene). After infecting B16 cells, resistance selection is performed with a concentration of 0.8 μg/mL of puromycin. Positive clones were confirmed and selected by Western blotting. The sgRNA sequences were listed in Table S2.

### Cell viability, migration, and scratch assay

Cells (B16, A875) were seeded at a density of 3 × 10^3^ cells per well in a 96-well plate. After adherence, different concentrations of ORY-1001 (RG-6016, Lollane, Shanghai, China) were added, and cell viability was assessed using the SRB method after 4 days of treatment.

Transwell culture inserts (TCS-002-024 JET, Guangzhou, China) were used for migration assays. The lower chamber was filled with DMEM medium containing 20% FBS. A serum-free DMEM medium containing approximately 8 × 10^3^ cells was added to the upper chamber. After 36 h, the cells on the lower surface of the polycarbonate membrane at the bottom of the chamber were fixed with 4% paraformaldehyde, stained with 0.5% crystal violet, and photographed for counting. Under the optical microscope, random fields of view were photographed at a magnification of 100×.

For the scratch assay, B16 and A875 cells were seeded into 6-well plates. Using a 200 μL pipette tip, uniform scratch wounds were made across the monolayer of cells. The wounded cells were then washed twice with PBS and maintained in serum-free culture medium. The width of the cell-free scratch was measured at 0 h and 36 h. The migration ability of cells was analyzed based on the healing area of the scratch.

### Total RNA extraction and quantitative real-time PCR

The Cell Total RNA Isolation Kit (Beibeibio, 082008, Zhengzhou, China) was used to obtain cellular RNA. To produce cDNA, RNA was reverse-transcribed using HiScript II Q Select RT SuperMix (Vazyme, R233-01, Nanjing, China). Real-time PCR mix (Vazyme, Q711, Nanjing, China) was used to detect the mRNA expression levels of the genes. The qPCR primers were purchased from Tsingke (Beijing, China). The qPCR primer sequences are shown in Table S3.

### Western blotting

Cells were collected and lysed using a RIPA buffer containing phosphatase inhibitors and protease inhibitors. Cell lysates were subjected to SDS-PAGE and subsequently electrotransferred to a PVDF membrane, which was incubated with the indicated primary antibodies, washed, and probed with HRP-conjugated secondary antibodies. The antibodies were detailed in Table S4. For the original, uncropped western blotting images, please refer to Supplementary information-original data.

### Chromatin immunoprecipitation

The ChIP assay was conducted using a ChIP assay kit (9003S, CST, USA). Control cells and B16 cells treated with 10 μM ORY-1001 were cross-linked with 1% formaldehyde for 15 min, and the chromatin DNA was digested with micrococcal nuclease. After immunoprecipitation, qPCR was performed to quantify the precipitated DNA. The percentage of enrichment was determined using the formula: % total = 2% × 2 ^(C[T]2%input-C[T]ChIP)^. The primers are listed in Tables S5–S7.

### Animal experiments

BALB/c-nude mice (5–6 weeks old, female) were obtained from Gempharmatech (Nanjing, China). We did two batches of animal experiments. In the first batch of experiments, twenty mice were randomly divided into four groups for the lung metastasis assay. Two groups were inoculated with 3 × 10^6^ B16 parental cells via the tail vein, and the other two groups were inoculated with 3 × 10^6^ OE YAP B16 cells via the tail vein. The treatment groups were given ORY-1001 solution (400 μg/kg, diluted in saline) every 7 days according to the body weight of mice, and the control groups were injected with an equal volume of saline. The mice were weighed every 3 days. The experiment was terminated after 3 weeks. The lung metastases of the mice were observed.

In the second batch of in vivo experiments, twenty mice were randomly divided into two groups for a lung metastasis assay. Two groups were inoculated with 3 × 10^6^ B16 parental cells via the tail vein, and the other two groups were inoculated with 3 × 10^6^ KO NF2 B16 cells via the tail vein. All other procedures were consistent with those in the first batch of experiments.

### Immunohistochemistry

Commercially available human melanoma tissue microarrays were purchased from Xi’an Zhongke Guanghua Bioaitech Co., Ltd. (K118Me01, Bioaitech, China). Formalin-fixed and paraffin-embedded melanoma tumor sample sections were taken to analyze by immunohistochemistry. Sections were deparaffinized in xylene and rehydrated in ethanol. After antigen retrieval and blocking, samples were stained with specified antibodies, and the results were scanned and subjected to quantitative analysis. The melanoma tissue microarray included 59 patients with duplicate samples per patient, consisting of 44 primary malignant melanomas, 12 metastatic malignant melanomas, and 3 normal skin tissues. Four samples (D9, D10, D11, D12) exhibited strong non-specific background staining and were therefore excluded from the quantitative analysis. Each patient’s tumor includes two tissue cores, with “*n*” representing the number of tissue cores. The clinical and pathological information of human melanoma samples were listed in Table S8. The antibodies used in immunohistochemistry are the same as those used in western blotting, and their information is listed in Table S4.

### Statistical analysis

All experiments were repeated three times independently. ImageJ software was used for quantitative analysis of the experimental results. Adobe Photoshop CS6 and Adobe Illustrator 2021 software were used for layout and integration of experimental images. GraphPad Prism 8.0 software was applied for data analysis. Mean comparisons between experimental groups were analyzed using the Student’s *t*-test, while multiple group comparisons were conducted using one-way analysis of variance (ANOVA). *P* value < 0.05 was considered statistically significant. (ns *P* > 0.05; ^*^P < 0.05; ^**^P < 0.01; ^***^P < 0.001; ^****^P < 0.0001).

## Results

### Targeting LSD1 inhibits melanoma cell migration

**Fig. 1:**
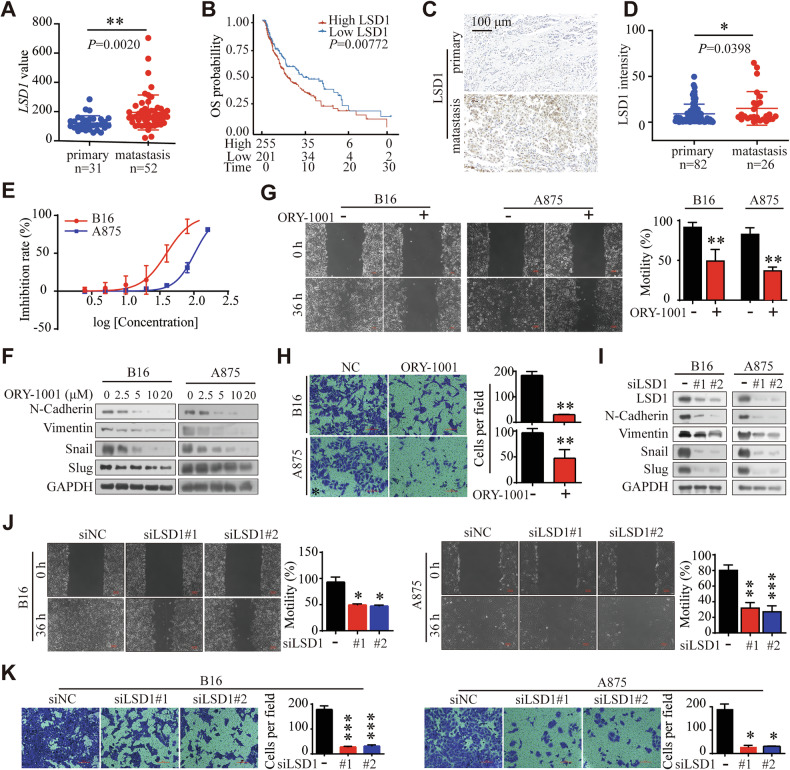
Targeting LSD1 impedes melanoma migration and metastasis. **A** LSD1 levels in tumor tissues from primary and metastasis patients from the Gene Expression Omnibus with the identifier GSE 8401. **B** Kaplan–Meier plotter analyses showing the overall survival of melanoma patients from The Cancer Genome Atlas (TCGA). **C** The staining of a human melanoma tissue microarray with the LSD1 antibody was performed. **D** Quantification of (**C**). **E** The inhibitory effect of ORY-1001 on the proliferation of B16 and A875 cells. **F** Inhibition of LSD1 by ORY-1001 affected the expression of EMT-related proteins. Inhibition of LSD1 by 10 μM ORY-1001 impaired the migratory ability of B16 and A875 cells detected by scratch tests (**G**) and transwell assays (**H**). **I** Inhibition of LSD1 by siRNA affected the expression of EMT-related proteins. Inhibition of LSD1 by siRNA impaired the migratory ability of B16 and A875 cells, as detected by scratch assays (**J**) and transwell assays (**K**). *P < 0.05, **P < 0.01, ***P < 0.001.

Based on our previous research, we have established that elevated LSD1 expression is associated with tumor metastasis. Furthermore, data from the Proteinatlas database (www.proteinatlas.org) indicate that LSD1 exhibits the highest expression levels among methylation modification enzymes in melanoma patients (Table 1). These findings prompted us to explore the role of LSD1 in melanoma metastasis further. By analyzing datasets from The Cancer Genome Atlas (TCGA), we discovered that LSD1 expression is significantly upregulated in tumor samples from patients with metastatic melanoma (Fig. 1A), and elevated LSD1 mRNA levels are correlated with poor prognosis (Fig. 1B). Furthermore, melanoma patient samples were analyzed by immunohistochemistry to evaluate the protein level of LSD1, revealing marked upregulation of LSD1 expression after the tumor metastasized (Fig. 1C, D). Based on the database (The Human Protein Atlas), we analyzed the correlation between LSD1 and EMT-related proteins in nine melanoma cells. The results showed that Vimentin and Slug were positively correlated with LSD1 (Supplementary Fig. 1A). Meanwhile, we also examined protein expression in six melanoma cells. Among them, B16-F10 is a highly metastatic subline of B16 obtained through in vitro screening. Compared with parental B16 cells, B16-F10 cells exhibited significantly higher expression levels of LSD1, YAP, and N-cadherin (Supplementary Fig. 1B). These results demonstrate that abnormal activation of LSD1 is connected with metastasis and poor survival in patients with melanoma.

**Table 1:** Gene expression levels (pTPM).

Gene	Level
DNMT3B	1.6
TET1	0.2
TET2	1.2
MeCP2	18.3
HDACs	0.6
SETD1A	11.5
DNMT3A	8.0
NSD1	8.0
NSD2	10.9
EZH2	8.6
SETD2	1.9
KDM1A	43.6
KDM5A	5.6
SETD1B	6.9

*pTPM* transcripts per million, *DNMT3B* DNA (cytosine-5)-methyltransferase 3B, *DNMT3A* DNA (cytosine-5)-methyltransferase 3A, *TET1* ten-eleven translocation protein 1, *TET2* ten-eleven translocation 2, *MeCP2* methyl-CpG-binding protein 2, *HDACs* histone deacetylases, *SETD1A* SET domain containing 1A, *SETD1B* SET domain containing 1B, *NSD1* nuclear receptor binding SET domain protein 1, *NSD2* nuclear receptor binding SET domain protein 2, *EZH2* enhancer of zeste homolog 2, *KDM1A* lysine-specific histone demethylase 1A, *KDM5A* lysine demethylase 5A.

To investigate the role of LSD1 in melanoma cells, we used the LSD1 inhibitor ORY-1001. ORY-1001 is a highly potent and selective KDM1A inhibitor that can form a covalent bond with the FAD cofactor, thereby affecting the formation of the LSD1 complex and promoting H3K4me2 accumulation on KDM1A target genes [24]. We employed the SRB experiment to assess the viability of A375, A875, and B16 cells following drug treatment. The IC50 values of ORY-1001 for A375, B16, and A875 cells are 31.11 ± 5.56, 40.18 ± 6.35, and 102.80 ± 6.10 μM, respectively (Fig. 1E; Supplementary Fig. 1C). These results suggest that ORY-1001 exhibits limited efficacy in inhibiting the proliferation of melanoma cells. Consequently, we next investigated the effect of ORY-1001 on the metastasis of melanoma cells. A375, A875, and B16 cells were treated with varying concentrations of ORY-1001, and the expression of EMT-related proteins was detected. It was found that ORY-1001 reduced the expression of N-cadherin, Vimentin, Snail, and Slug proteins in a concentration-dependent manner (Fig. 1F; Supplementary Fig. 1D–F). To further evaluate the effect of ORY-1001 on cell migration, the scratch assay was used to detect the changes in the scratch damage repair ability of melanoma cells. The results showed that, compared with the control group, ORY-1001-treated cells exhibited a smaller cell scratch injury healing area and reduced migration ability (Fig. 1G). Similarly, Transwell experiments confirmed that ORY-1001 significantly impaired the migration capacity of melanoma cells (Fig. 1H). Consistent with these findings, knockdown of LSD1 using siLSD1 also significantly inhibits the expression of EMT-related proteins and inhibits the migration in B16 and A875 cells (Fig. 1I–K, Supplementary Fig. 1G). These results indicate that inhibition of LSD1, either pharmacologically or genetically, effectively suppresses the metastatic potential of melanoma cells.

### Targeting LSD1 inhibits melanoma cell migration through downregulating YAP expression

YAP, a tumor-promoting factor downstream of the Hippo pathway, plays an important role in tumor initiation, invasion, and metastasis [25]. In a separate study conducted by our team, the inhibition of LSD1 is associated with the concurrent downregulation of YAP during malignant progression in lung cancer [26]. Thus, this prompted us to investigate whether YAP mediates LSD1-driven melanoma metastasis.

**Fig. 2:**
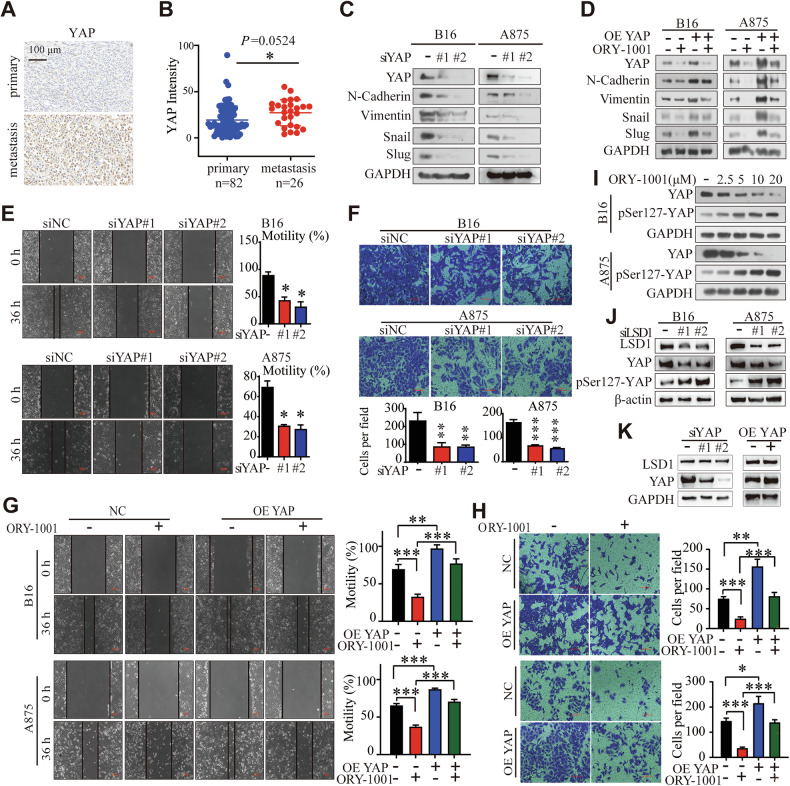
YAP accounts for the inhibitory effects of targeting LSD1 on melanoma migration. **A** Immunohistochemical analysis of YAP in melanoma from patients. **B** Quantification of (**A**). **C** The levels of EMT-related proteins were detected following YAP knockdown. **D** YAP was overexpressed in B16 and A875 cells, and the cells were treated with 10 μM ORY-1001. The levels of EMT-related proteins were evaluated by western blotting. After YAP was knocked down, the cell migration ability was detected by scratch assay (**E**) and transwell assay (**F**). The scratch assay (**G**) and the transwell assay (**H**) were used to detect the changes in the migration ability of cells after the overexpression of YAP and treatment with 10 μM ORY-1001. **I** The effect of different concentrations of ORY-1001 on YAP protein levels. **J** The expression of YAP was detected by western blotting after LSD1 was knocked down. **K** The expression of LSD1 was detected by western blotting after YAP was knocked down. ns *P* > 0.05, *P < 0.05, **P < 0.01, ***P < 0.001.

Firstly, we evaluate the role of YAP in melanoma metastasis. We investigated the expression of YAP in patients by immunohistochemical analysis; the results showed obvious enhancement of YAP in metastasis samples (Fig. 2A, B). To clarify the role of YAP in melanoma metastasis, we knocked down YAP or ectopically overexpressed YAP (OE YAP) in B16 and A875 cells (Fig. 2C, D; Supplementary Fig. 2A, B). The results showed that YAP knockdown significantly reduced the protein expression of N-cadherin, Vimentin, Snail, and Slug in B16 and A875 cells (Fig. 2C; Supplementary Fig. 2A), whereas YAP overexpression had the opposite effect (Fig. 2D; Supplementary Fig. 2B). Similarly, compared to the control group, YAP knockdown resulted in a smaller scratch injury healing area and a weaker migratory ability (Fig. 2E, F), while YAP overexpression enhanced cell motility (Fig. 2G, H). The above results indicate that YAP expression significantly controls melanoma cell migration.

Secondly, to investigate whether the LSD1 inhibitor ORY-1001 affects melanoma metastasis through YAP, we examined its effect on YAP protein expression in melanoma cells. The results revealed that ORY-1001 increased YAP phosphorylation and reduced YAP protein expression in A375, A875, and B16 cells in a concentration-dependent manner (Fig. 2I; Supplementary Fig. 2C; Supplementary Fig. 3A). Similarly, knockdown of LSD1 also downregulated YAP expression (Fig. 2J; Supplementary Fig. 3B). In contrast, neither YAP knockdown nor YAP overexpression altered LSD1 expression levels (Fig. 2K; Supplementary Fig. 3C). These results support that LSD1 regulates YAP protein abundance in our study. We investigated further whether YAP overexpression could counteract the regulatory effect of LSD1 on melanoma cell migration. The results showed that YAP overexpression reversed the inhibitory effect of ORY-1001 on the expression of EMT-related proteins in melanoma cells (Fig. 2D). More interestingly, YAP overexpression completely restored the scratch wound-healing capacity and migratory ability suppressed by ORY-1001 (Fig. 2G, H). The above results suggest that LSD1 regulates melanoma cell migration by modulating YAP expression.

### Targeting LSD1 decreases YAP expression through activating the Hippo pathway

**Fig. 3:**
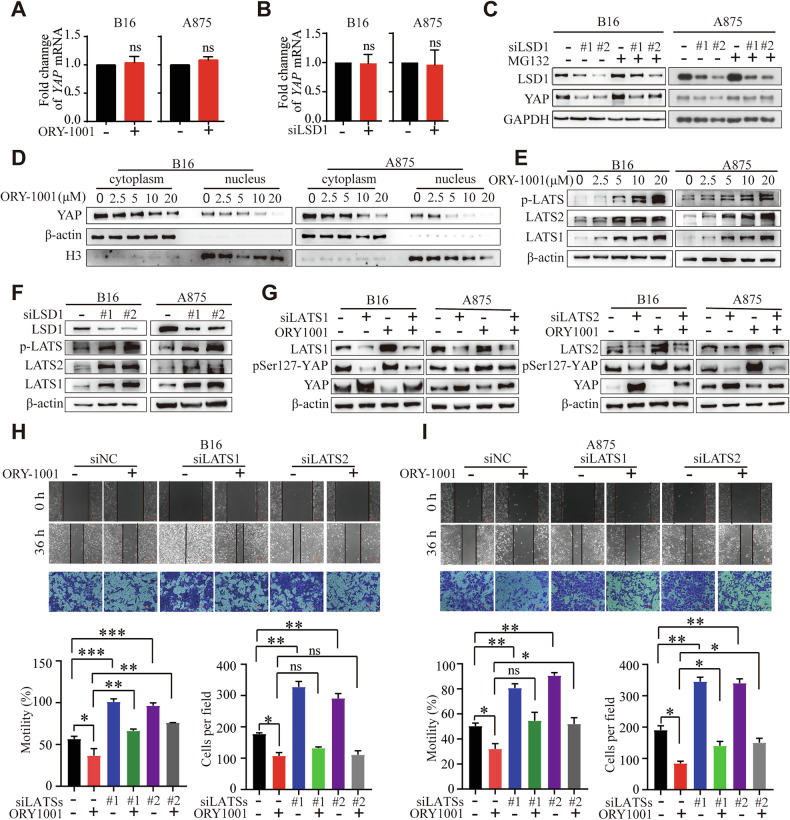
Targeting LSD1 inhibits YAP expression through the Hippo pathway. **A** The *YAP* mRNA level in 10 μM ORY-1001-treated and control cells was detected by qRT-PCR. **B** *YAP* mRNA levels in LSD1 knockdown and control cells were detected by qRT-PCR. **C** After knocking down LSD1 in B16 and A875 cells, the proteasome inhibitor MG132 (40 μM) was added for 6 h, and then the total protein was extracted to detect the expression of YAP. **D** The nuclear-cytoplasmic separation experiment was used to detect the YAP expression levels in the nucleus after the action of different concentrations of ORY-1001. **E** After treating B16 and A875 cells with ORY-1001 in gradient concentrations, the expressions of LATS1, LATS2, and p-LATS proteins were detected by western blotting. **F** After knockdown of LSD1 in B16 and A875 cells, the protein expression levels of LATS1, LATS2, and p-LATS were assessed by western blotting. **G** After knocking down LATS1/2 in B16 and A875 cells, ORY-1001 at a concentration of 10 μM was applied to these two cell lines, and the levels of YAP and p-YAP proteins were detected by western blotting. The migration abilities of **H** B16 and **I** A875 cells with LATS1/2 knockdown or ORY-1001 treatment were assessed using a scratch assay and a transwell assay. ns *P* > 0.05, *P < 0.05, **P < 0.01, ***P < 0.001.

qPCR analysis showed that knockdown or inhibition of LSD1 did not alter YAP mRNA levels in B16 and A875 cells (Fig. 3A, B), indicating that LSD1 does not regulate YAP expression at the transcriptional level. It is reported that phosphorylated YAP binds to the 14-3-3 protein and undergoes degradation via the proteasome pathway in the cytoplasm, which ultimately decreases the nuclear translocation of YAP. Therefore, MG132 was used to detect the stability of YAP by western blotting. The results showed that knocking down LSD1 significantly reduced YAP protein expression (Fig. 3C; Supplementary Fig. 4A). Furthermore, treatment with MG132 significantly accumulated LSD1 and YAP protein, an effect that was mitigated by the use of siLSD1 (Fig. 3C; Supplementary Fig. 4A). These findings suggest that LSD1 inhibition may promote YAP phosphorylation and subsequent proteasomal degradation. To further explore the effect of LSD1 on YAP nuclear translocation, we conducted the nuclear-cytoplasmic separation experiment. The results demonstrated that ORY-1001 significantly reduced YAP expression in the nucleus (Fig. 3D; Supplementary Fig. 4B), indicating that ORY-1001 promotes YAP phosphorylation and degradation, thereby limiting its nuclear entry.

YAP is primarily regulated by the Hippo pathway, serving as the main transcriptional effector downstream of this pathway [27]. When the Hippo pathway is activated, the LATS1 and LATS2 are phosphorylated, triggering a kinase cascade that promotes the phosphorylation and degradation of YAP [28]. Next, we further explored whether ORY-1001 regulates YAP phosphorylation by activating the Hippo pathway. The results showed that ORY-1001 increased the protein levels of LATS1, LATS2 and p-LATS (the antibody simultaneously recognizes LATS1 phosphorylated at Thr1079 and LATS2 phosphorylated at Thr1041) in a concentration-dependent manner (Fig. 3E; Supplementary Fig. 4C). Similar results were obtained by knocking down LSD1 (Fig. 3F; Supplementary Fig. 4D). In addition, ORY-1001 increased p-YAP expression while decreasing total YAP expression, effects that were reversed by the knockdown of LATS1/2 (Fig. 3G; Supplementary Fig. 5A). Furthermore, ORY-1001 significantly inhibited the cell migration ability, which was partially reversed by the knockdown of LATS1/2 (Fig. 3H, I). These findings collectively indicate that targeting LSD1 regulates YAP expression and melanoma cell migration through the activation of the Hippo pathway, thereby suppressing melanoma cell migration.

### Targeting LSD1 promotes LATS1/2 transcriptional activation

**Fig. 4:**
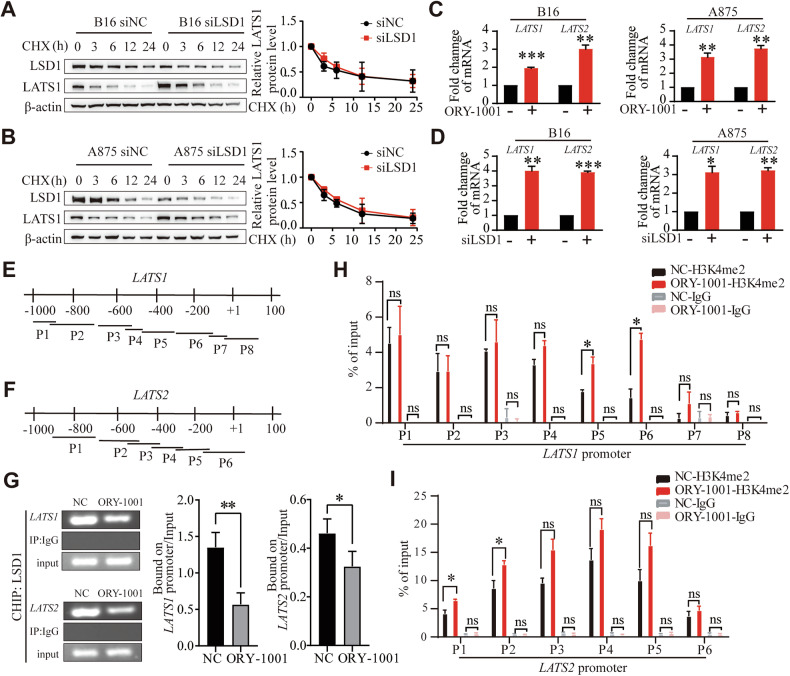
Targeting LSD1 modulates the Hippo pathway through augmenting H3K4me2 in the LATS1/2 promoter region. **A** After LSD1 was knocked down in B16 cells, the expression of LATS1 was detected by western blotting after the addition of protein synthesis inhibitor (CHX, 30 ng/mL) for different times. **B** After LSD1 was knocked down in A875 cells, the expression of LATS1 was detected by western blotting after the addition of protein synthesis inhibitor (CHX, 30 ng/mL) for different times. **C** The mRNA levels of *LATS1* and *LATS2* in B16 and A875 cells were detected by qRT-PCR after treatment with 10 μM ORY-1001 for 96 h. **D** The mRNA levels of *LATS1* and *LATS2* in cells after LSD1 knockdown were detected by qRT-PCR. **E** The primers for the *LATS1* promoter region. **F** The primers for the *LATS2* promoter region. **G** After treatment with 10 μM ORY-1001 for 96 h in B16 melanoma cells, a ChIP assay was carried out using an anti-LSD1 antibody, and the anti-IgG antibody was introduced as a negative control. The protein-chromatin immunoprecipitates were subjected to PCR analyses using LATS1/2 primers. Quantitative ChIP studies were conducted to characterize the enrichment of H3K4me2 at promoters of *LATS1* (**H**) and *LATS2* (**I**) in B16 cells treated with or without 10 μM ORY-1001 for 96 h. ns *P* > 0.05, *P < 0.05, **P < 0.01, ***P < 0.001.

The above findings indicate that the Hippo pathway is a critical mechanism through which LSD1 regulates YAP expression and melanoma cell migration. Given that inhibition of LSD1 significantly increased the protein levels of LATS1 and LATS2 (Fig. 3E, F), we further investigated the regulatory effects of LSD1 on the expression of LATS1 and LATS2. As shown in Fig. 4A, B, knockdown of LSD1 in B16 and A875 cells significantly increased LATS1 protein level without altering its half-life. qRT-PCR analysis showed that the mRNA level of *LATS1/2* were upregulated following LSD1 inhibition or knockdown (Fig. 4C, D), indicating that LSD1 inhibition promotes the transcriptional activation of LATS1 and LATS2. These results collectively indicate that LSD1 regulates the expression of LATS1 at the transcription level.

LSD1, a flavin adenine dinucleotide (FAD)-dependent demethylase, specifically removes monomethyl and dimethyl modifications at H3K4 and H3K9 [29] to modulate gene transcription [30, 31]. We performed ChIP-qPCR to verify the epigenetic regulation of LATS1 and LATS2 by LSD1. Primers were designed spanning from −1000 to +400 bp around the transcription start sites of *LATS1* and *LATS2* genes (Fig. 4E, F). Firstly, ChIP-qPCR using an LSD1-specific antibody confirmed that LSD1 directly binds to the promoter regions of LATS1 and LATS2. Notably, treatment with ORY‑1001 significantly reduced the enrichment of LSD1 at these promoters compared with the control group (Fig. 4G). Then, given that H3K4 dimethylation is generally associated with active gene modification [32], and previous studies have reported that ORY-1001 inhibits demethylase activity, leading to the accumulation of H3K4me2 at the promoter regions of target genes [33], ChIP-qPCR experiments using an H3K4me2-specific antibody were performed to determine whether LSD1 directly affects H3K4me2 levels in the *LATS1/2* promoter region. As shown in Fig. 4H, I (raw CT values are provided in Tables S9 and S10), compared with the control group, DNA enrichment in the *LATS1* promoter region (-473 bp, -122 bp) and the *LATS2* promoter region (-878 bp, -534 bp) was significantly increased in cells treated with the LSD1 inhibitor. These results showed that LSD1 inhibition promotes the modification of H3K4me2 in the *LATS1/2* promoter region, leading to their transcriptional activation.

### Targeting LSD1 regulates the Hippo pathway through upregulated NF2 expression

**Fig. 5:**
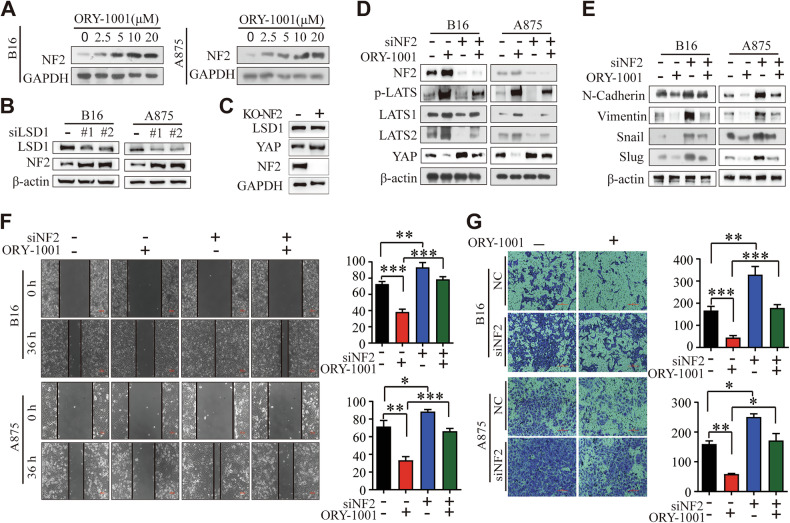
Targeting LSD1 activates an upstream factor of the Hippo pathway, known as NF2/Merlin. **A** Detect NF2 protein expression after treating B16 and A875 cells with different concentrations of ORY-1001. **B** Knockdown LSD1 in B16 and A875 cells, and assess the expression of NF2 protein using western blotting. **C** The protein levels of LSD1 and YAP were assessed by western blotting in KO NF2 B16 cells. After knocking down NF2 in B16 and A875 cells, 10 μM ORY-1001 was applied to these two cell lines, and the expression of **D** Hippo pathway-related proteins and **E** EMT-related proteins was detected by western blotting. **F** The migration ability of B16 and A875 cells with NF2 knockdown or 10 μM ORY-1001 treatment was detected by a scratch assay. **G** The migration ability of melanoma cells with NF2 knockdown or 10 μM ORY-1001 treatment was detected by a transwell assay. ns *P* > 0.05, *P < 0.05, **P < 0.01, ***P < 0.001.

The NF2 (Merlin) protein, encoded by the tumor suppressor gene *NF2*, has been reported to act as an upstream activator of the Hippo pathway [34]. *NF2* deficiency leads to the abnormal activation of developmental pathways, thereby facilitating tumor development and malignant progression [35]. Therefore, we explored whether NF2 plays a role in the regulation of the Hippo-YAP pathway by LSD1. NF2 protein levels were detected in A375, A875and B16 cells treated with different concentrations of ORY-1001. The results indicate that ORY-1001 increased NF2 protein expression in a concentration-dependent manner (Fig. 5A; Supplementary Fig. 6A, B). Similarly, knockdown of LSD1 also upregulated NF2 protein expression (Fig. 5B; Supplementary Fig. 6C). On the contrary, although knockout of NF2 increased the expression of YAP, it did not affect the expression level of LSD1 (Fig. 5C; Supplementary Fig. 6D). These results demonstrate that LSD1 expression is not regulated by NF2 in melanoma cells.

To further explore the role of NF2 in activating the Hippo pathway mediated by the LSD1 inhibitor, we transfected B16 and A875 cells with siNF2 and detected changes in Hippo pathway-related proteins. The results showed that ORY-1001 significantly activated the Hippo pathway, as evidenced by the upregulation of p-LATS protein expression and the downregulation of YAP protein expression (Fig. 5D). However, knockdown of NF2 inhibited the activation of the Hippo pathway and significantly reversed the effect of ORY-1001 on the Hippo pathway and YAP expression (Fig. 5D; Supplementary Fig. 6E). These results indicate that the LSD1 inhibitor suppresses YAP expression by activating the Hippo pathway with NF2. Next, we explored the influence of siNF2 on the regulation of EMT-related markers by LSD1. As depicted in Fig. 5E (Supplementary Fig. 6F), in comparison to the control group, knockdown of NF2 resulted in an upregulation of N-cadherin, Vimentin, Snail, and Slug, and reversed the inhibitory effect of ORY-1001 on these EMT markers in melanoma cells. We then evaluated the cell migration ability using a wound-healing assay and a transwell cell migration experiment. The results revealed that knockdown of NF2 significantly promoted cell migration and counteracted the inhibitory effects of the LSD1 inhibitor in melanoma migration (Fig. 5F, G). The above results demonstrate that ORY-1001 regulates the Hippo pathway by upregulating NF2 expression, thereby inhibiting YAP expression and suppressing melanoma cell migration.

### Targeting LSD1 promotes NF2 transcriptional activation

**Fig. 6:**
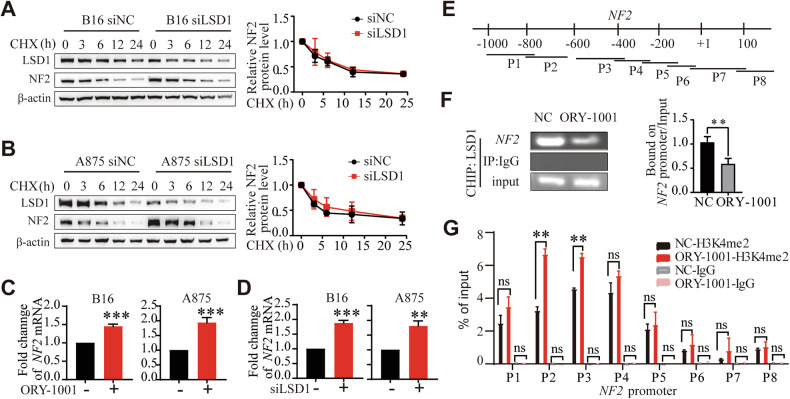
Targeting LSD1 promotes NF2 transcriptional activation. **A** After LSD1 was knocked down in B16 cells, the expression of LATS1 was detected by western blotting after the addition of protein synthesis inhibitor (CHX, 30 ng/mL) for different times. **B** After LSD1 was knocked down in A875 cells, the expression of LATS1 was detected by western blotting after the addition of protein synthesis inhibitor (CHX, 30 ng/mL) for different times. **C** The mRNA levels of *NF2* in B16 and A875 cells were detected by qRT-PCR after treatment with 10 μM ORY-1001 for 96 h. **D** The mRNA level of *NF2* in cells after LSD1 knockdown was detected by qRT-PCR. **E** The primers for the *NF2* promoter region. **F** B16 melanoma cells were treated with 10 μM ORY-1001. After 96 h, the B16 cells were harvested and applied to a CHIP assay using an anti-LSD1 antibody. The protein-chromatin immunoprecipitates were subjected to PCR analyses using the *NF2* primers. **G** Quantitative ChIP studies were conducted to characterize the enrichment of H3K4me2 at promoters of the *NF2* gene in B16 cells treated with 10 μM ORY-1001 for 96 h. ns *P* > 0.05, **P < 0.01, ***P < 0.001.

Subsequently, we explored the regulatory role of LSD1 on Merlin by using the protein synthesis inhibitor CHX. As shown in Fig. 6A, B, knockdown of LSD1 significantly increased Merlin protein level without altering its half-life. We also observed an increase in the mRNA level of Merlin following LSD1 inhibition or knockdown (Fig. 6C, D). These results indicate that LSD1 inhibition promotes Merlin expression at the transcriptional level. To confirm whether LSD1 directly binds to the promoter region of *NF2* and modulates H3K4me2 level, we conducted the ChIP experiment with the LSD1 antibody and the H3K4me2 antibody, separately. Primers were designed in the region from −1000 to +400 bp of the transcription start site of the *NF2* gene (Fig. 6E). ChIP-qPCR experiments using an LSD1-specific antibody showed that ORY-1001 inbibited the binding of LSD1 to the promoter regions of LATS1/2 (Fig. 6F). ChIP-qPCR experiments using an H3K4me2-specific antibody revealed that, compared to the control group, DNA enrichment was significantly increased in the regions (−820 bp, −683 bp) and (−520 bp, −382 bp) of the *NF2* promoter in cells treated with the LSD1 inhibitor (Fig. 6G and Table S11). These results indicate that the LSD1 inhibition promotes H3K4me2 modification in the *NF2* promoter region, leading to its transcription activation.

### LSD1 regulates melanoma metastasis through the NF2-Hippo-YAP axis in vivo

**Fig. 7:**
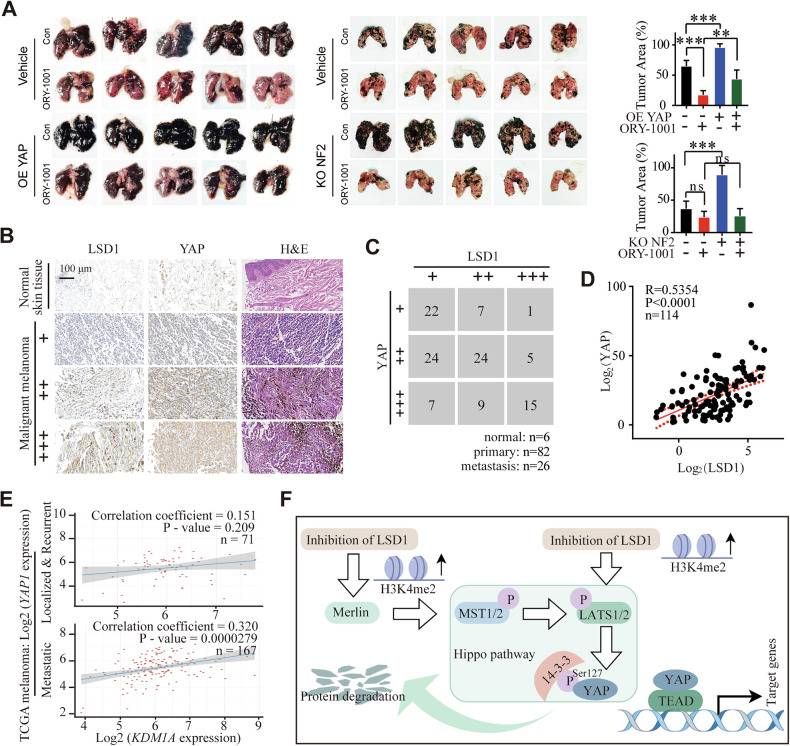
LSD1 regulates melanoma metastasis through the NF2-Hippo-YAP axis in vivo. **A** Inject different groups of cells into BALB/c-nude mice via the tail vein at a dose of 3 × 10^6^ cells/200 μL. After successful establishment of the model, begin administering ORY-1001 (400 μg/kg) with an interval of 7 days between doses. After 3 weeks, sacrifice the mice and observe the metastasis of lesions in the organs. **B** Specimens were collected from 54 melanoma patients and 3 normal skin tissues, and immunohistochemical staining of LSD1 and YAP was processed. **C** Expression levels of LSD1 and YAP exhibited a statistically significant positive association in melanoma patients. **D** The correlation analyses between YAP and LSD1 were performed. **E** The correlation between *KDM1A* and *YAP* expression was also examined in melanoma patients from the TCGA database. **F** A model of ORY-1001 regulating melanoma metastasis through Merlin/NF2 and the Hippo pathway.

Having confirmed that siNF2, siLATS1, and overexpression of YAP promote the migration of melanoma cells and reverse the inhibitory effect of ORY-1001 in vitro, we sought to validate these findings in vivo. Lung metastasis models were established using *NF2* knockout (KO NF2) and YAP overexpressing (OE YAP) B16 cells. As shown in Fig. 7A, compared to the vehicle group, ORY-1001 significantly inhibited the lung metastasis of melanocytes. However, in mice injected with cells overexpressing YAP or lacking NF2, lung metastasis of melanoma cells was significantly enhanced, and these manipulations markedly reversed the inhibitory effect of ORY‑1001 on lung metastasis. The above results indicate that ORY-1001 inhibits lung metastasis of melanoma in vivo, and activating the NF2-Hippo pathway to down-regulate the expression of YAP is one of the main factors.

To investigate the clinical relevance of LSD1 and YAP in patients with melanoma, the expression of LSD1 and YAP in melanoma patients was detected by immunohistochemical analysis. As shown in Fig. 7B, C, the results demonstrated a statistically significant positive correlation between YAP and LSD1 protein levels in melanoma patient samples, including both primary and metastatic tumors (*R* = 0.5354, *P* < 0.0001). Positive correlations were revealed among the levels of YAP and LSD1 (Fig. 7D). Interestingly, in an independent clinical sample group from TCGA, the association between *KDM1A* and *YAP* has also been verified. As shown in Fig. 7E, in tumor tissues from 167 patients with metastatic melanoma, *KDM1A* and *YAP* are positively correlated (Correlation coefficient = 0.320, *P* value = 0.0000279). However, in tumor tissues from patients with localized and recurrent melanoma, *KDM1A* and *YAP* expression are not correlated (*P* value = 0.209). These findings collectively support that LSD1 regulates melanoma metastasis through the NF2-Hippo-YAP axis.

## Discussion

Melanoma is a highly aggressive tumor with a propensity for metastasis. Despite advancements in chemotherapy, targeted therapy, and immunotherapy, metastatic melanoma often develops resistance to these treatments, resulting in low response rates and poor overall survival. Therefore, finding new therapeutic targets and drugs remains critical for improving outcomes in metastatic melanoma. This study is the first to discover that LSD1 is closely related to melanoma metastasis. We found that targeting LSD1 significantly inhibits the migration and metastasis of melanoma cells both in vivo and in vitro. In terms of molecular mechanisms, inhibiting LSD1 indirectly upregulates NF2 expression and directly increases the Hippo pathway kinases LATS1/2 expression to activate the Hippo pathway, leading to the phosphorylation and degradation of the downstream transcriptional regulator YAP. This cascade ultimately suppresses melanoma cell migration and metastasis.

LSD1 mediates epigenetic reprogramming related to EMT in mouse hepatocytes, thereby promoting cell migration and chemoresistance [36]. It also promotes EMT, migration, and proliferation in NSCLC and hepatocellular carcinoma [37]. Additionally, LSD1 interacts with Slug to promote the migration and invasion of breast cancer cell lines [38, 39]. These findings position LSD1 as a potential therapeutic target for preventing tumor migration. Our study further demonstrates that the LSD1 inhibitor regulates EMT-related proteins and inhibits melanoma cell migration. The migration processes in melanoma are regulated by various mechanisms. For example, the neural cell adhesion molecule (NCAM) regulates EMT and migration through the Src/Akt/mTOR/cofilin signaling pathway [40], while Polo-like kinase 1 (PLK1) activates NOTCH signaling to promote EMT [41]. Here, our research confirms that LSD1 inhibition of melanoma cell migration is mediated by downregulating YAP.

YAP, a key oncogenic effector, is frequently overactivated in tumors, driving tumor cell stemness, proliferation, drug resistance, and metastasis [42–45]. Studies have shown that YAP induces the EMT process in NSCLC by interacting with the transcriptional coactivator TEAD to regulate the transcription of Slug, thereby promoting the invasion and migration [46]. When the Hippo pathway is activated, upstream kinases are phosphorylated and activated, which in turn triggers a series of kinase cascade reactions [47], ultimately leading to YAP phosphorylation, cytoplasmic retention, and proteasomal degradation. In this study, we confirmed the promoting role of YAP in the migration of melanoma cells, which is consistent with existing studies showing that YAP promotes the metastasis of NSCLC and breast cancer. LSD1 inhibition promotes YAP phosphorylation and degradation, preventing its nuclear translocation and thereby inhibiting melanoma migration.

In the Hippo pathway, LATS1/2 serves as a central switch by integrating upstream signals and limiting the oncogenic activity of YAP/TAZ [48]. Under physiological conditions, the activity of YAP/TAZ is mainly restricted by LATS1/2 kinases, but dysregulation of the Hippo pathway is frequently observed in tumors, where it contributes to tumorigenesis and progression. For example, peptidyl-prolyl cis-trans isomerase 1 (PIN1) interacts with STK3, inducing its ubiquitination and degradation, which inactivates the Hippo pathway and activates YAP, driving oncogenic signals and the occurrence of melanoma [49]. Our study finds that pharmacological or genetic inhibition of LSD1 both activates the Hippo signaling pathway and downregulates YAP, but LATS1/2 knockdown reverses this process. Migration experiments further confirmed that LATS1/2 knockdown accelerates the migration of melanoma and reverses the inhibitory effects of the LSD1 inhibitor. These studies indicate that the Hippo pathway is involved in the regulation of LSD1 in melanoma cell migration. Molecularly, ChIP-qPCR demonstrates that the LSD1 inhibitor enhances H3K4me2 modification in the promoter region of LATS1/2, thereby promoting their transcriptional activation. In this study, no conserved motifs or shared features were identified in the LSD1-bound promoter regions of LATS1 and LATS2, suggesting that the transcription factors mediating LSD1 recruitment to these specific targets remain unidentified and warrant further investigation. Dysregulation of the Hippo pathway, characterized by YAP/TAZ activation and LATS1/2 inactivation, is common in various cancers, including liver, colon, breast, and oral cancers [50]. Further research should explore whether LSD1 inhibitors can also inhibit metastasis in these tumors.

NF2 regulates growth arrest in response to cell confluence, which has been confirmed in multiple studies [51]. It has been reported that Merlin shuttles between the nucleus and cytoplasm [52], and in the nucleus, it binds to the E3 ubiquitin ligase CRL4DCAF1 through its N-terminal FERM domain to inhibit its activity [53]. NF2 deficiency has been shown to activate YAP through the Hippo pathway, triggering epithelial cell migration [54]. In *NF2*-mutated tumors, such as mesothelioma, activated CRL4^DCAF1^ induces LATS1/2 ubiquitination and degradation, leading to YAP/TAZ activation and tumorigenesis [55]. Our study explored the correlation between LSD1 and NF2 in melanoma and revealed that LSD1 inhibition upregulates the expression of NF2, while NF2 knockdown inhibits the activation of the Hippo pathway. This is consistent with previous findings that NF2 knockdown significantly reduces the phosphorylation of LATS1/2 and Hippo pathway activation [56]. ChIP-qPCR experiments further found that LSD1 inhibitors promote the transcriptional activation of *NF2*. In vivo studies confirmed that NF2 knockout, LATS1/2 knockout, and YAP overexpression reverse the inhibition of ORY-1001 on melanoma metastasis. Therefore, LSD1 epigenetically regulates the expression level of NF2, thereby affecting the activation of the Hippo pathway in melanoma cells.

Interestingly, a previous study reported that YAP/TAZ promotes polyamine biosynthesis in mouse livers, which in turn causes the hydroxylation of the eukaryotic translation factor 5A (eIF5A), thereby facilitating the efficient translation of LSD1 [21]. In sharp contrast, our data indicate that LSD1 acts as an upstream epigenetic regulatory factor of YAP in melanoma. Specifically, pharmacological inhibition of LSD1 activates the NF2-Hippo pathway, leading to YAP phosphorylation, cytoplasmic retention, and degradation. Notably, our regulatory model is consistent with recent findings in triple-negative breast cancer, where LSD1 was shown to epigenetically sustain YAP1 expression by regulating H3K4 methylation of METTL14 and subsequently blocking the degradation of YAP mediated by YTHDF2 [57]. Whether LSD1 also modulates YAP activity through Hippo-independent mechanisms in melanoma warrants further investigation.

In summary, this study demonstrates for the first time that LSD1 regulates melanoma cell migration and metastasis through the NF2-Hippo-YAP signaling pathway. The LSD1 inhibition upregulates NF2 and LATS1/2 to activate the Hippo pathway, leading to YAP phosphorylation and degradation, ultimately inhibiting melanoma cell migration and metastasis (Fig. 7F). These findings not only deepen our understanding of the molecular mechanisms underlying melanoma metastasis but also provide a new theoretical foundation for the development of LSD1 inhibitors and targeted therapies for melanoma.

## Supplementary information

Supplementary Fig. 1. LSD1 is associated with the metastasis of melanoma cells.

Supplementary Fig. 2. YAP is involved in targeting LSD1 to affect the metastasis of melanoma cells.

Supplementary Fig. 3. YAP is involved in targeting LSD1 to affect the metastasis of melanoma cells.

Supplementary Fig. 4. Targeting LSD1 activates the Hippo pathway.

Supplementary Fig. 5. Targeting LSD1 activates the Hippo pathway.

Supplementary Fig. 6. Targeting LSD1 activates the Hippo signaling pathway by upregulating NF2 expression.

Supplementary figure legends

Table S1. siRNA sequences.

Table S2. sgRNA sequences.

Table S3. qPCR primer sequences.

Table S4. Antibody.

Table S5. LATS1 primer sequences.

Table S6. LATS2 primer sequences.

Table S7. NF2 primer sequences.

Table S8. Detailed clinicopathological information for the 59 human melanoma samples.

Table S9. Anti-H3K4me2 ChIP (IgG control) followed by PCR using LATS1 primers.

Table S10. Anti-H3K4me2 ChIP (IgG control) followed by PCR using LATS2 primers.

Table S11. Anti-H3K4me2 ChIP (IgG control) followed by PCR using NF2 primers.

original data

## Data Availability

The dataset generated and/or analyzed during the current study is available from the corresponding author upon reasonable request.
