# Supplementary figures and images for: Targeting lysine-specific demethylase 1 inhibits melanoma metastasis via the NF2-Hippo-YAP pathway
Source: Cell Death Dis. 2026 May 16;17(1):626. doi: 10.1038/s41419-026-08872-1 (PMC13346636; doi:10.1038/s41419-026-08872-1)

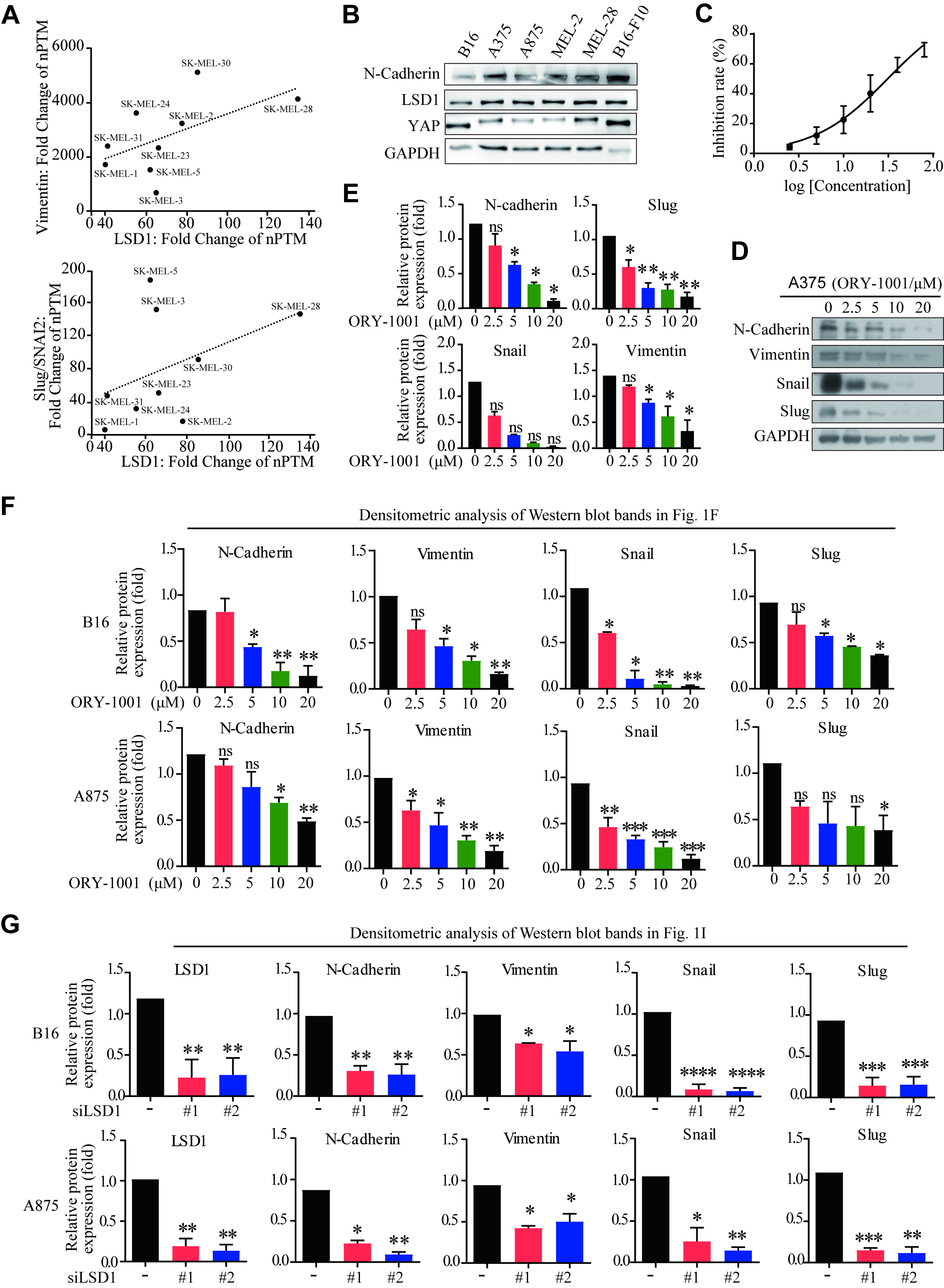

Supplement: Supplementary file 1 — Supplementary Fig. 1. LSD1 is associated with the metastasis of melanoma cells. [file 41419_2026_8872_MOESM1_ESM.tif]

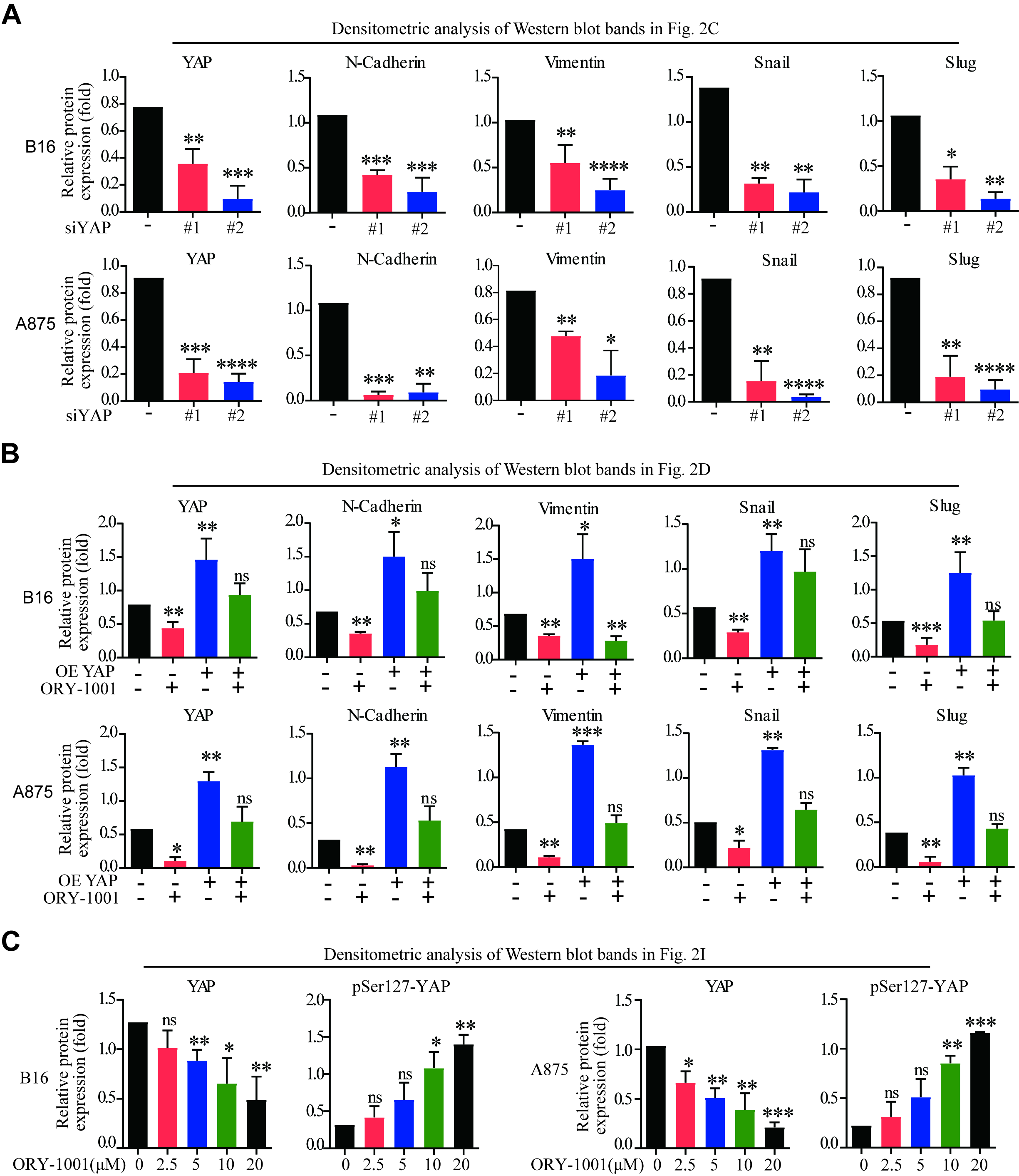

Supplement: Supplementary file 2 — Supplementary Fig. 2. YAP is involved in targeting LSD1 to affect the metastasis of melanoma cells. [file 41419_2026_8872_MOESM2_ESM.tif]

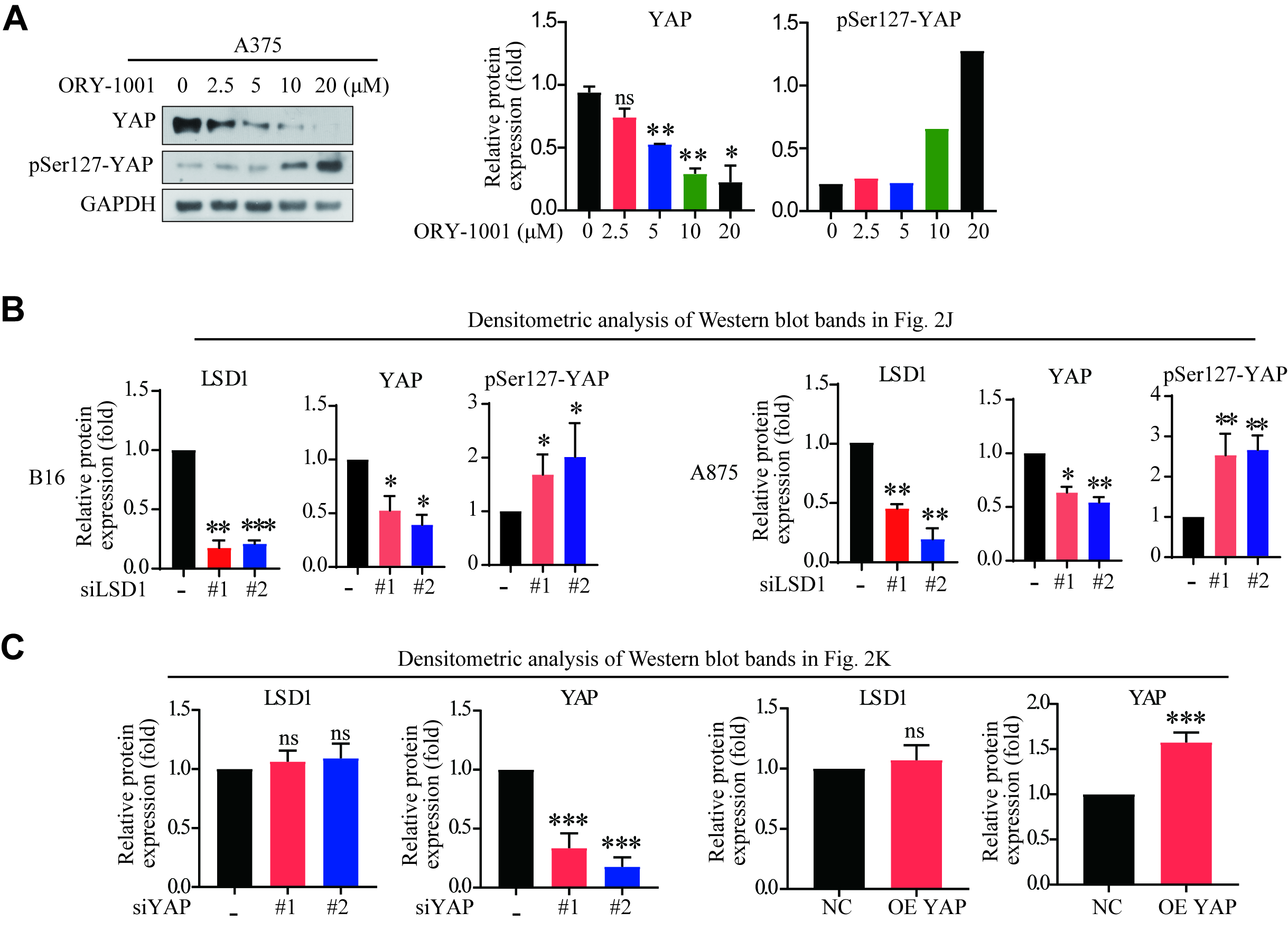

Supplement: Supplementary file 3 — Supplementary Fig. 3. YAP is involved in targeting LSD1 to affect the metastasis of melanoma cells. [file 41419_2026_8872_MOESM3_ESM.tif]

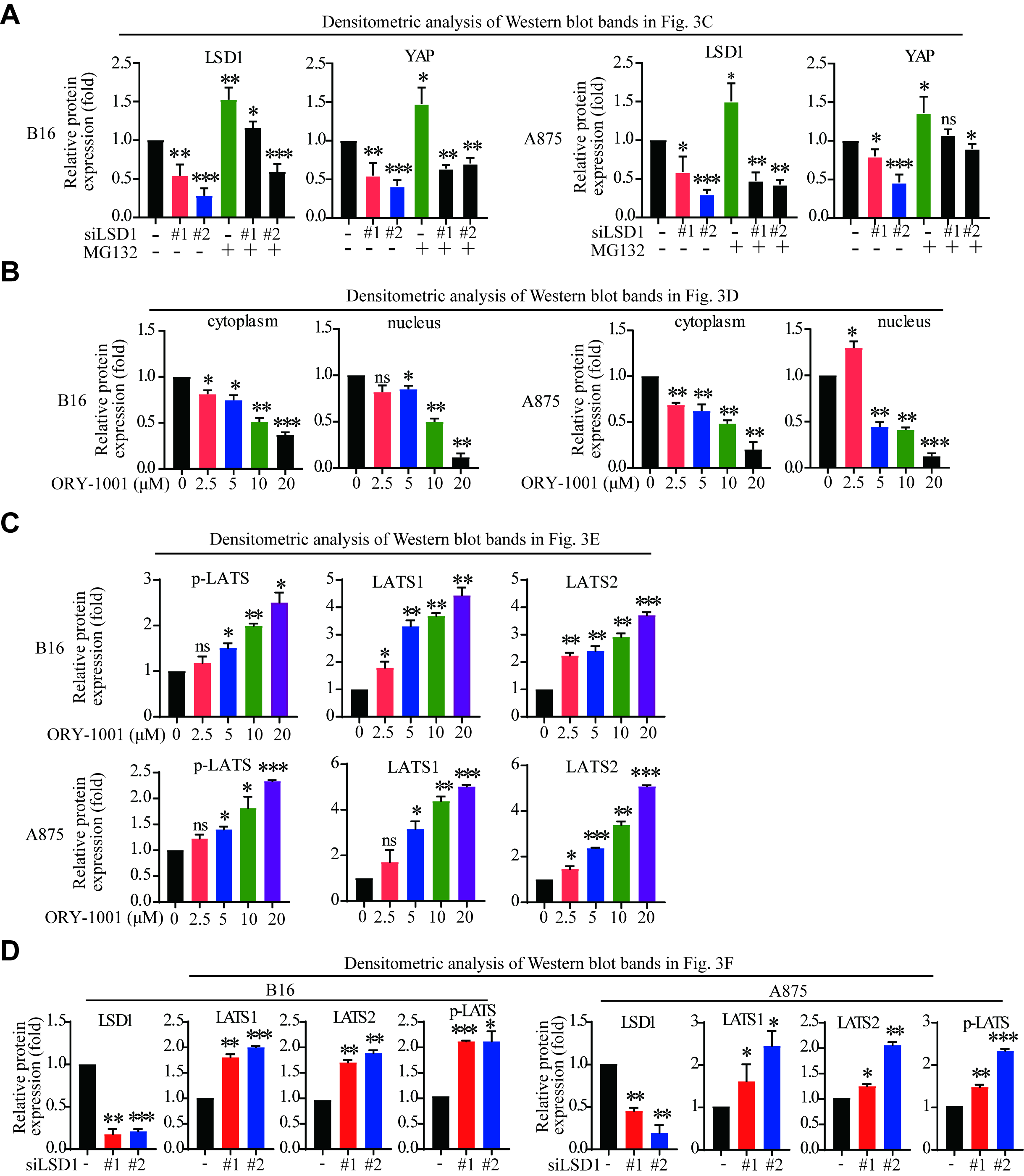

Supplement: Supplementary file 4 — Supplementary Fig. 4. Targeting LSD1 activates the Hippo pathway. [file 41419_2026_8872_MOESM4_ESM.tif]

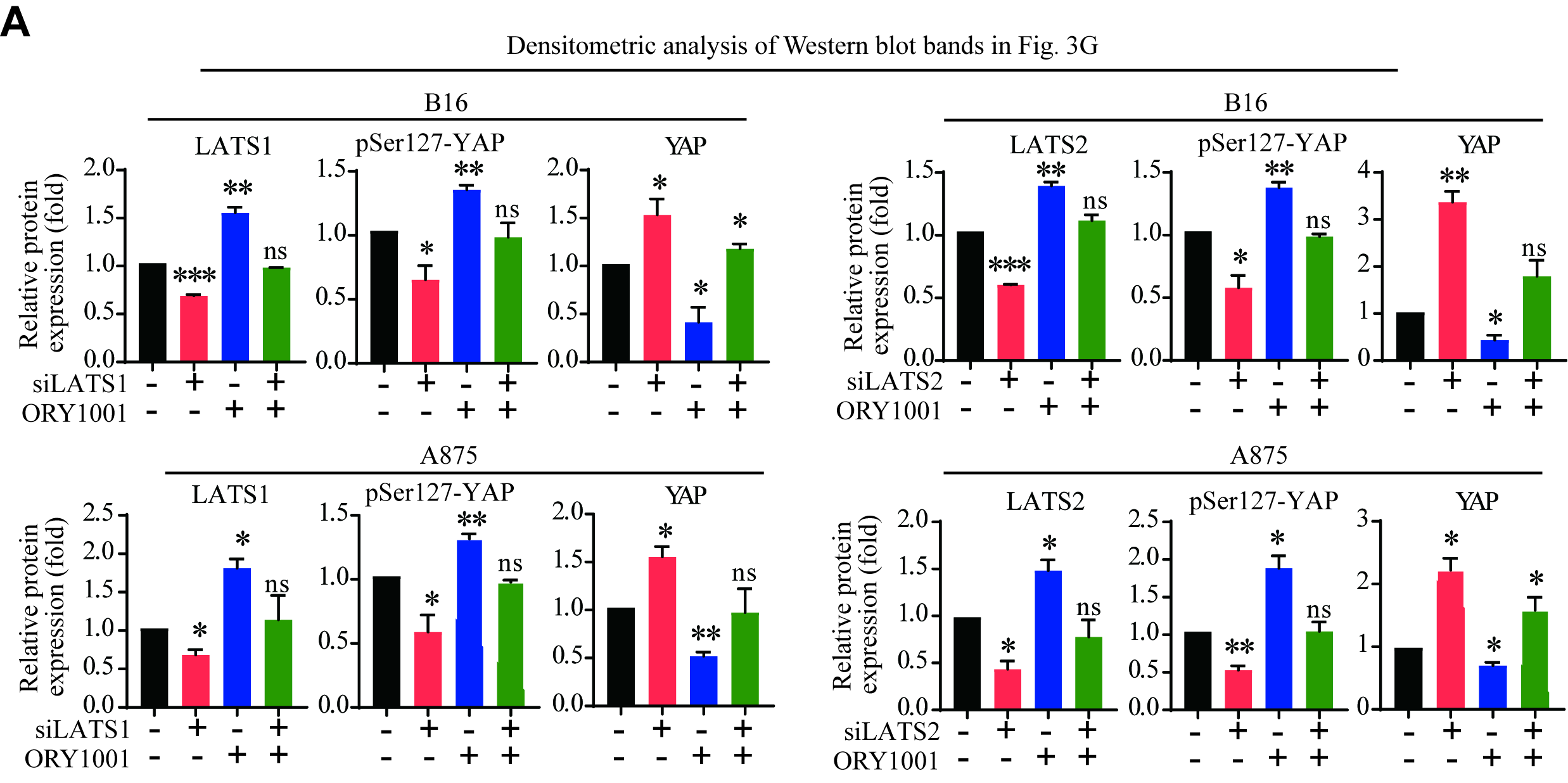

Supplement: Supplementary file 5 — Supplementary Fig. 5. Targeting LSD1 activates the Hippo pathway. [file 41419_2026_8872_MOESM5_ESM.tif]

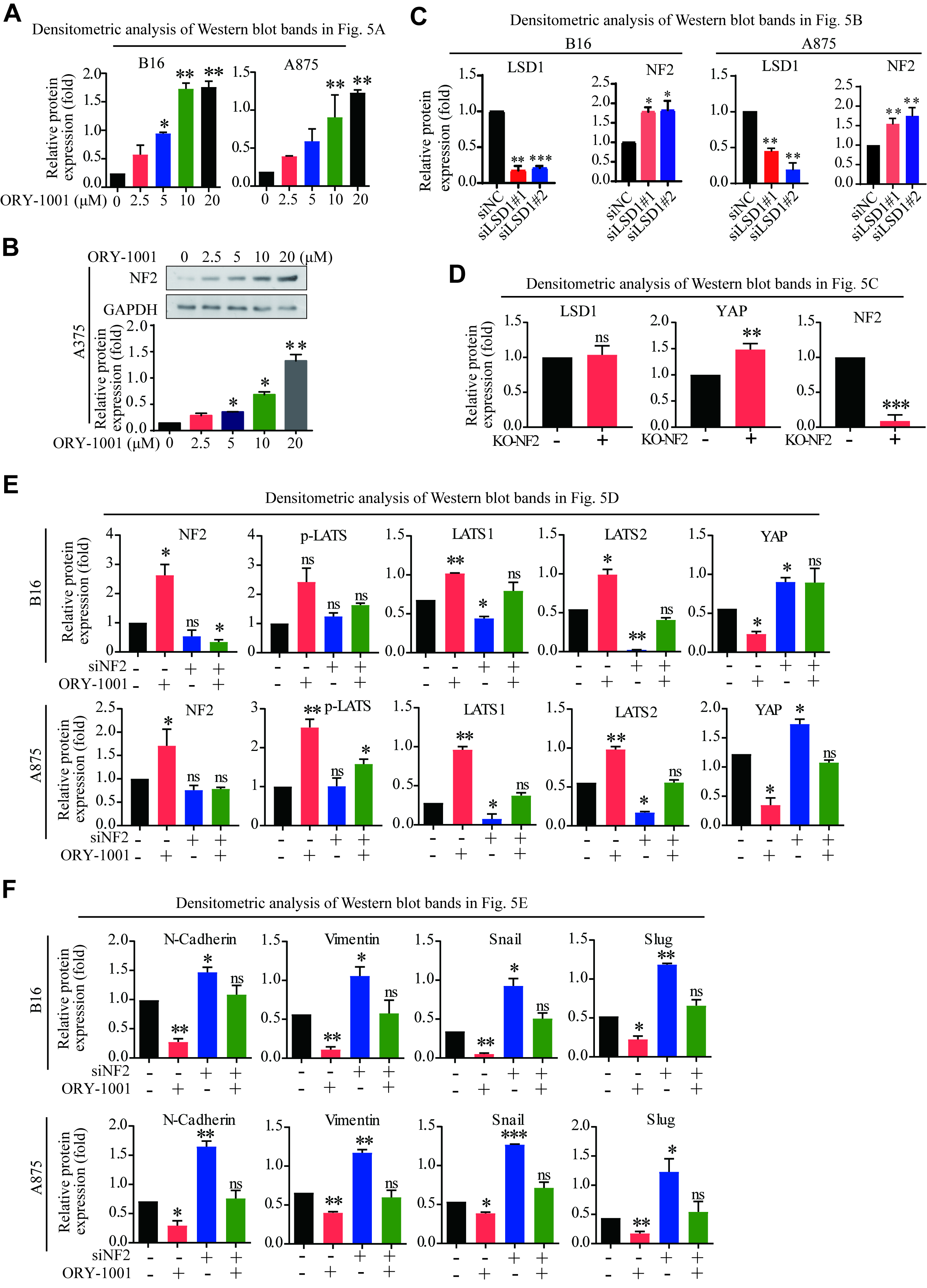

Supplement: Supplementary file 6 — Supplementary Fig. 6. Targeting LSD1 activates the Hippo signaling pathway by upregulating NF2 expression. [file 41419_2026_8872_MOESM6_ESM.tif]
