# Supplementary material for: Targeting lysine-specific demethylase 1 inhibits melanoma metastasis via the NF2-Hippo-YAP pathway
Source: Cell Death Dis. 2026 May 16;17(1):626. doi: 10.1038/s41419-026-08872-1 (PMC13346636; doi:10.1038/s41419-026-08872-1)
Supplement: Supplementary file 7 — Supplementary figure legends [file 41419_2026_8872_MOESM7_ESM.docx]

**Supplementary Fig. 1. LSD1 is associated with the metastasis of melanoma cells.** (A) RNA expression data was obstained as normalized transcript per million (nTPM) values of cancer cell lines (https://www.proteinatlas.org). The correlation between LSD1 and EMT-related proteins was analyzed in nine melanoma cell lines. (B) The protein levels of N-Cadherin, LSD1 and YAP were assessed by western blotting in six melanoma cell lines. (C) The inhibitory effect of ORY-1001 on the proliferation of A375 cells. (D-E) Inhibition of LSD1 by ORY-1001 affected the expression of EMT-related proteins in A375 cells. (D) Western blotting was used to detecting the protein expression of EMT-related proteins in A375 cells. (E) The quantification of (D). Densitometric analysis of Western blot bands in Fig. 1F (F) and Fig. 1F (G).

**Supplementary Fig. 2. YAP is involved in targeting LSD1 to affect the metastasis of melanoma cells.** (A) Quantification of Western blot signals in Fig. 2C. (B) Quantification of Western blot signals in Fig. 2D. (C) Quantification of Western blot signals in Fig. 2I.

**Supplementary Fig. 3.** **YAP is involved in targeting LSD1 to affect the metastasis of melanoma cells.** (A) The effect of different concentrations of ORY-1001 (96 h) on YAP protein levels in A375 cell lines. (B) Relative protein levels were determined by densitometry in Fig. 2J. (C) Relative protein levels were determined by densitometry in Fig. 2K.

**Supplementary Fig. 4. Targeting LSD1 activates the Hippo pathway.** Gray value analysis of Western blot bands in Fig. 3C-3F. (A) Fig. 3C. (B) Fig. 3D. (C) Fig. 3E. (D) Fig. 3F.

**Supplementary Fig. 5.** **Targeting LSD1 activates the Hippo pathway.** (A) Densitometric quantification of protein bands in Fig. 3G.

**Supplementary Fig. 6. Targeting LSD1 activates the Hippo signaling pathway by upregulating NF2 expression.** (A) Gray value analysis of Western blot bands in Fig. 5A. (B) NF2 protein expression was assessed in A375 cells treated with varying concentrations of ORY-1001 (96 h). (C-F) Gray value analysis of Western blot bands in Fig. 5B-5E. (C) Fig. 5B. (D) Fig. 5C. (E) Fig. 5D. (F) Fig. 5E.
