# Supplementary material for: Targeting lysine-specific demethylase 1 inhibits melanoma metastasis via the NF2-Hippo-YAP pathway
Source: Cell Death Dis. 2026 May 16;17(1):626. doi: 10.1038/s41419-026-08872-1 (PMC13346636; doi:10.1038/s41419-026-08872-1)

Fig 1F


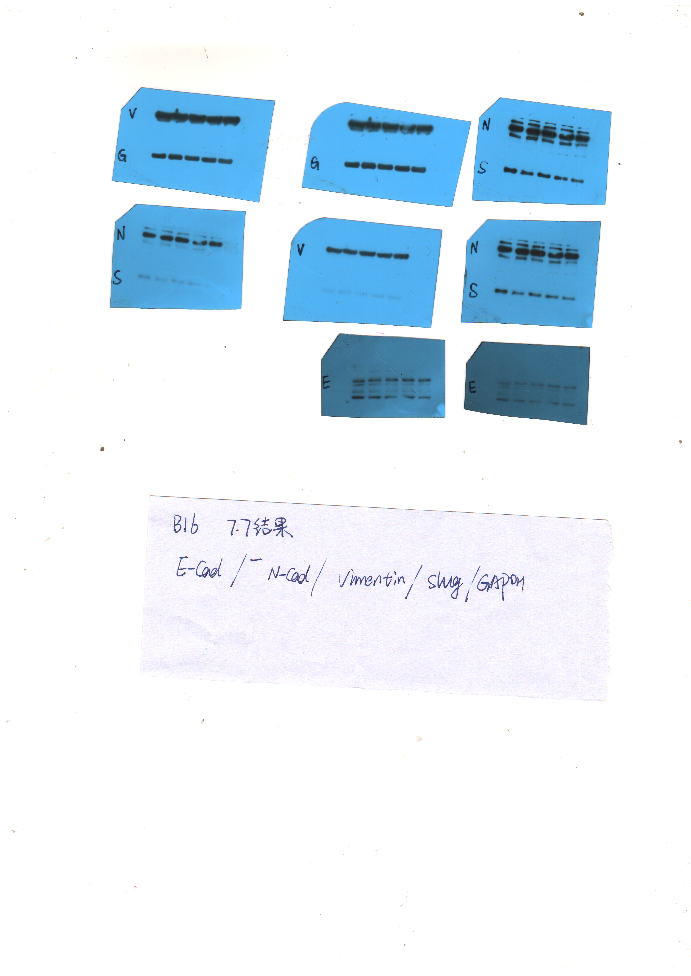

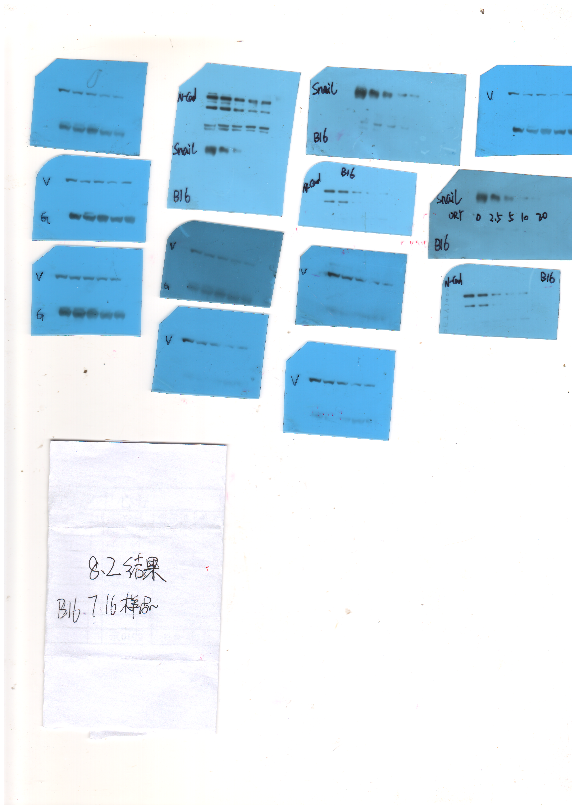

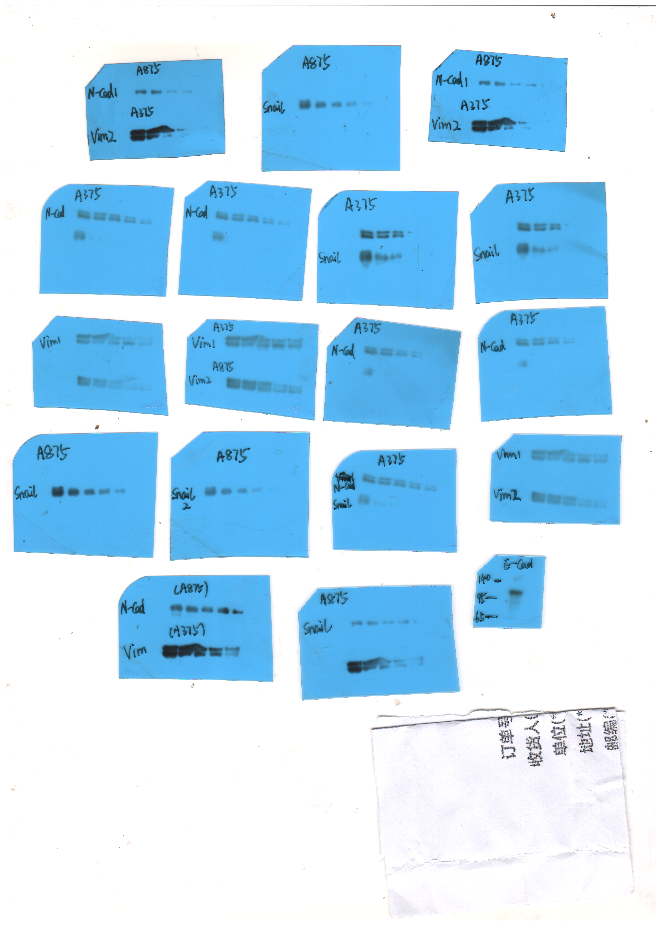

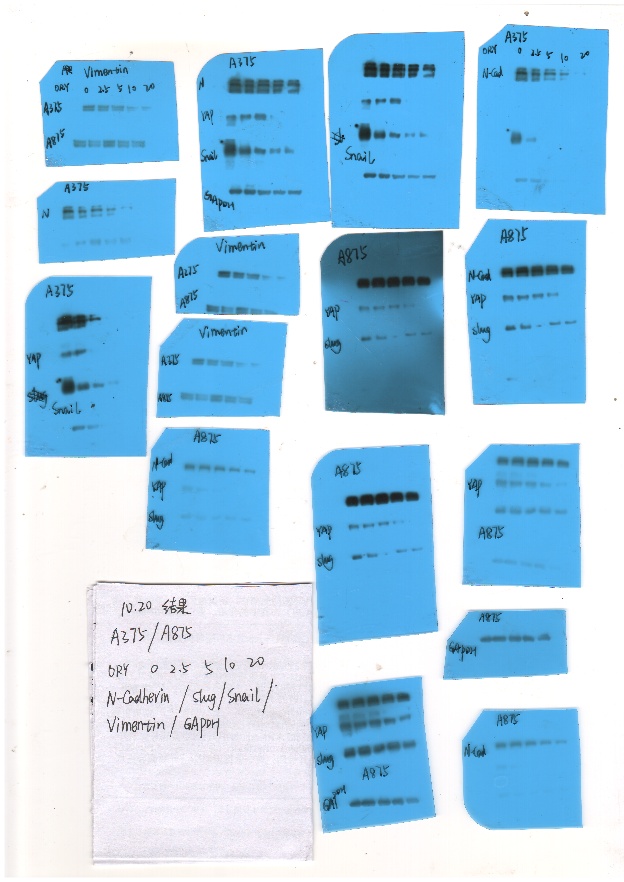

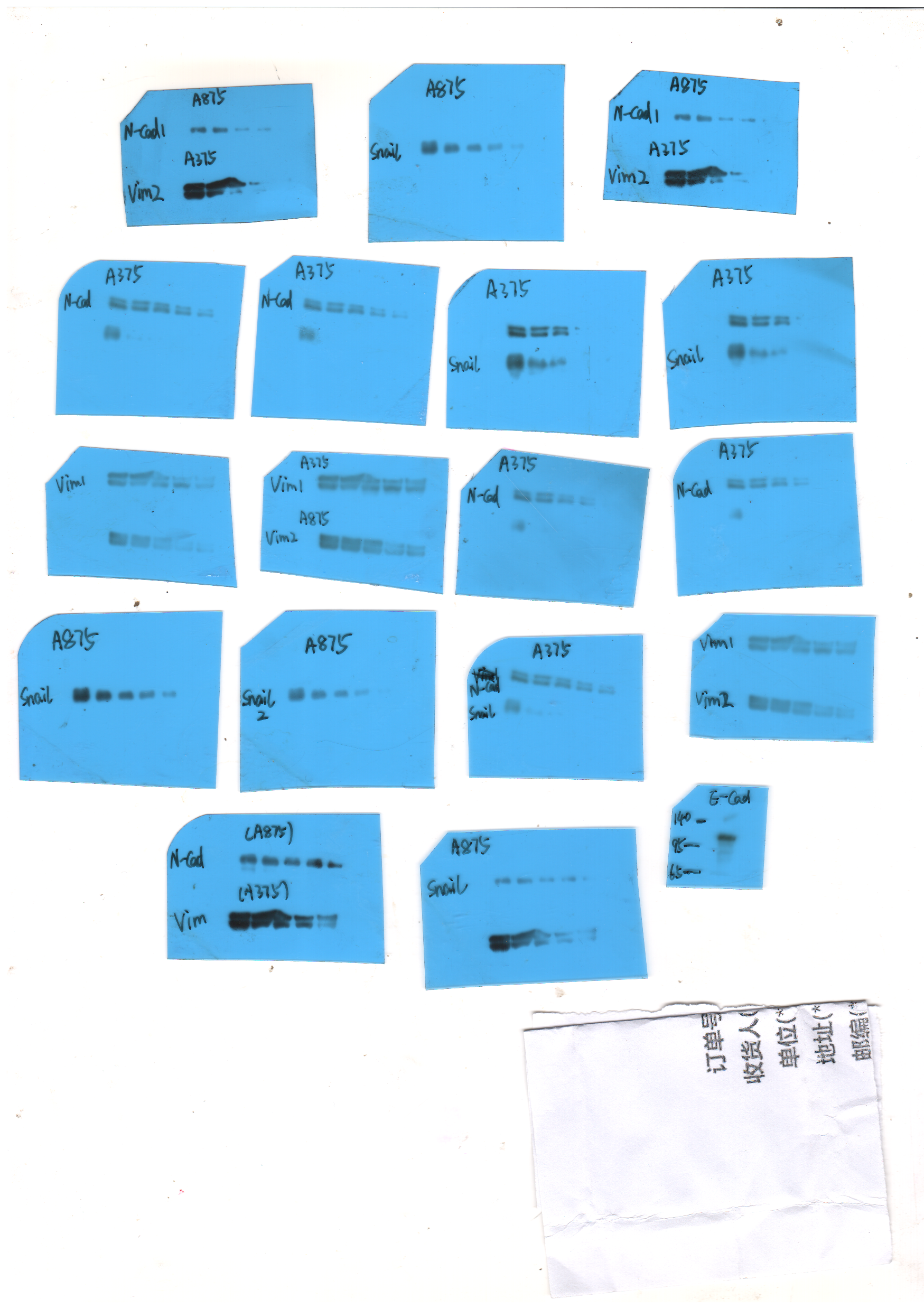

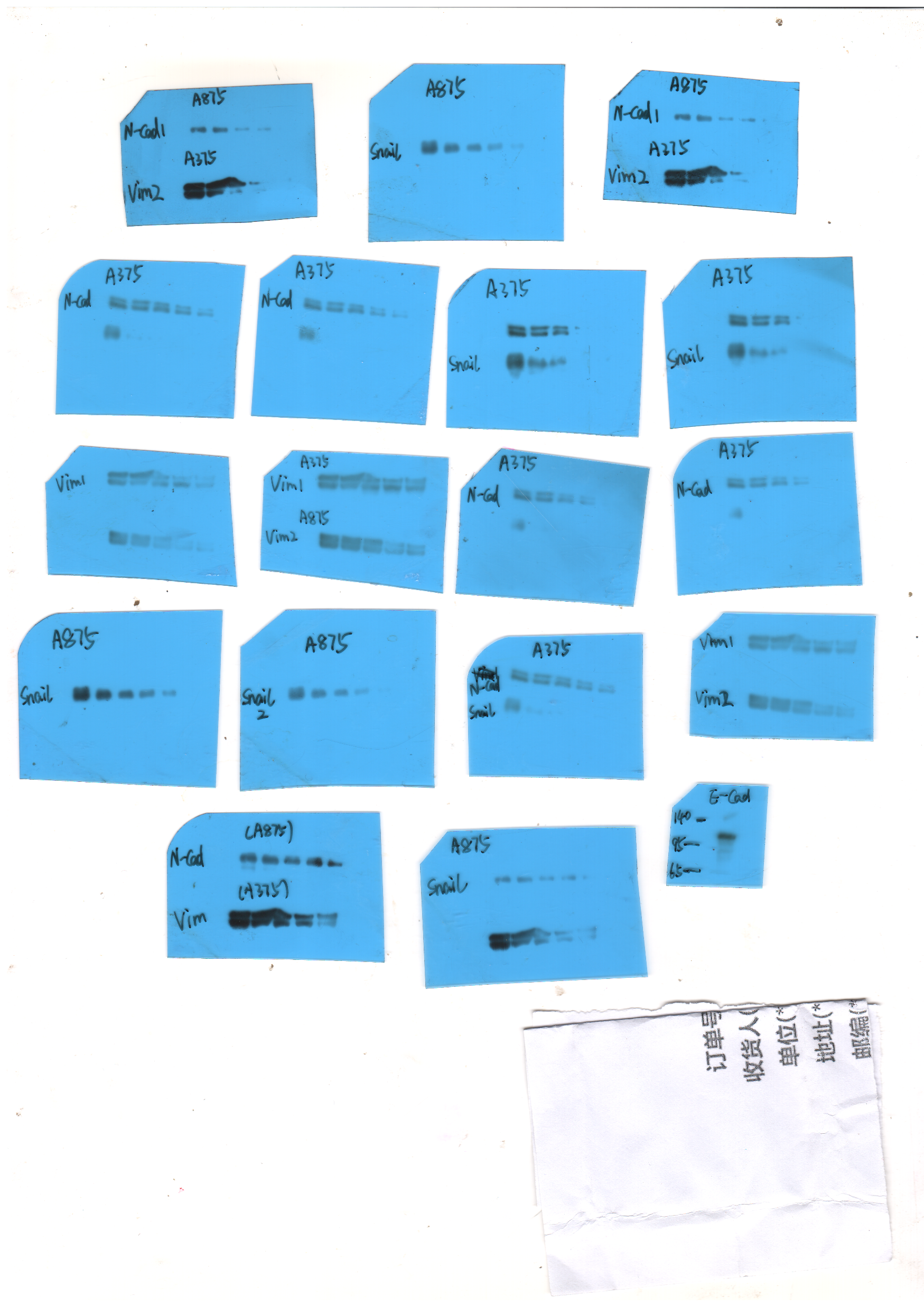

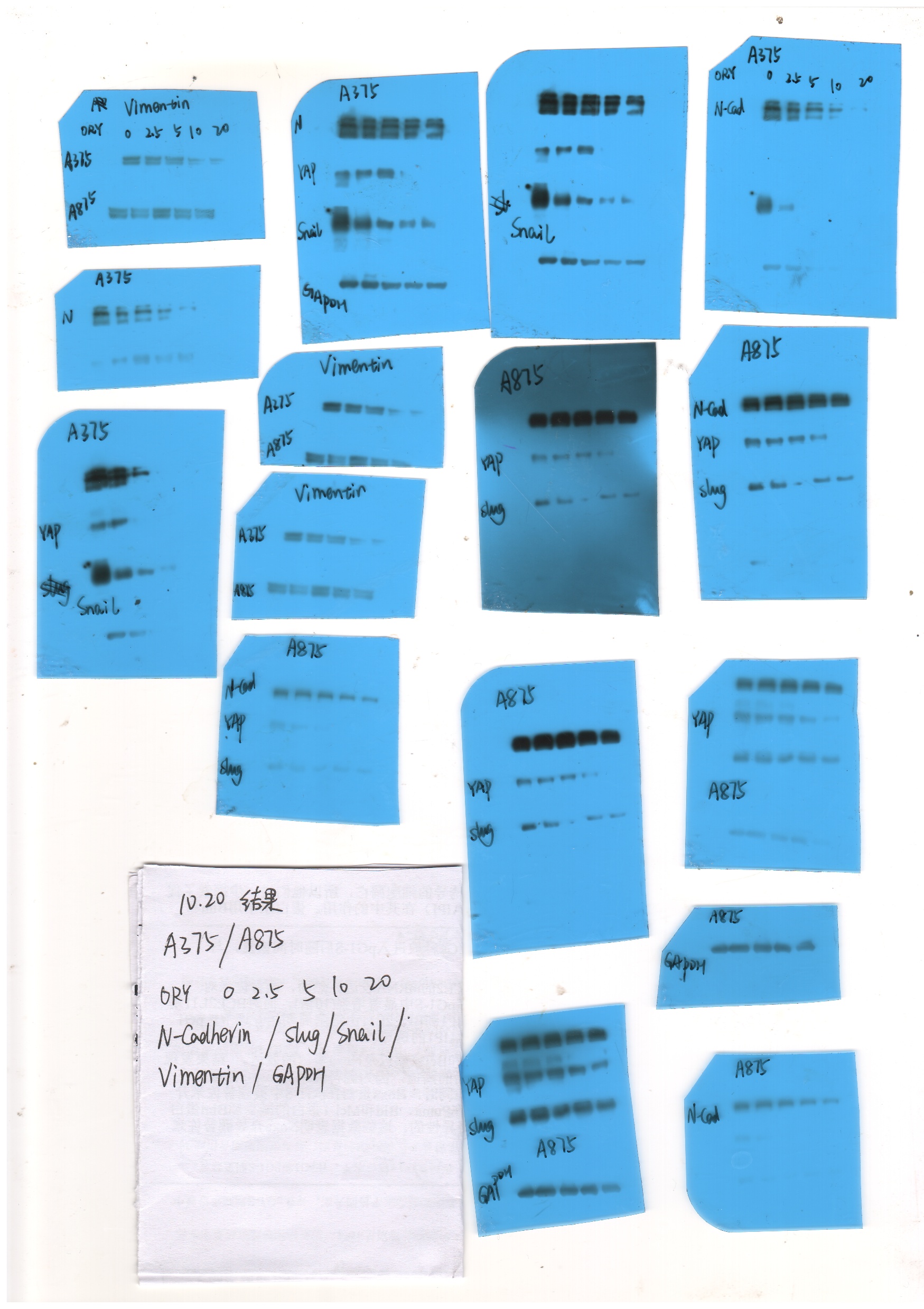

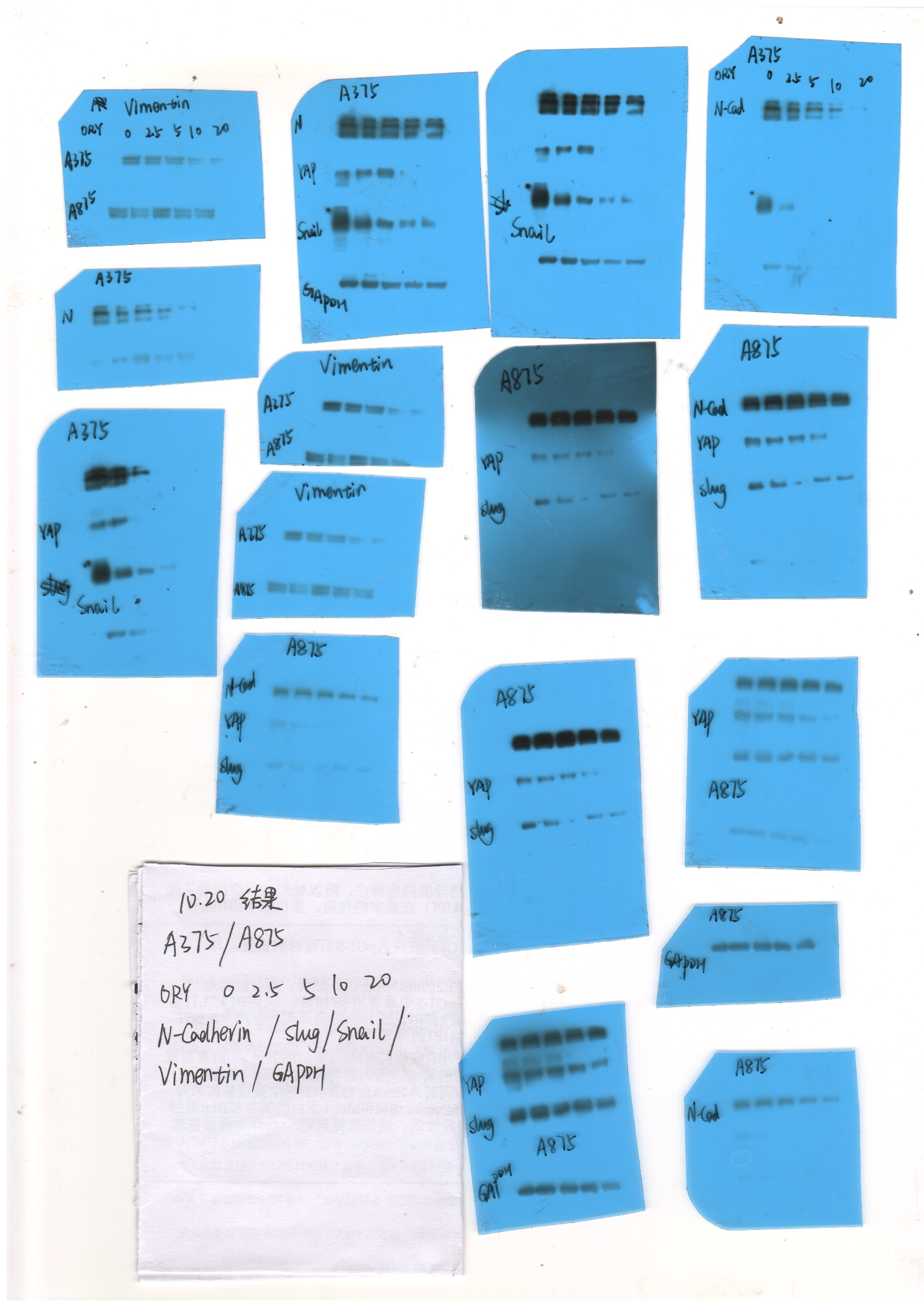


Fig 1I


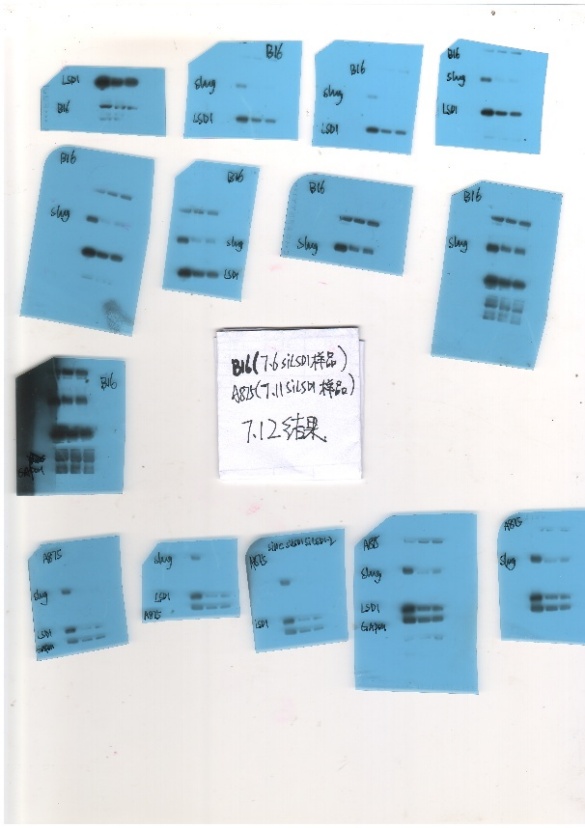

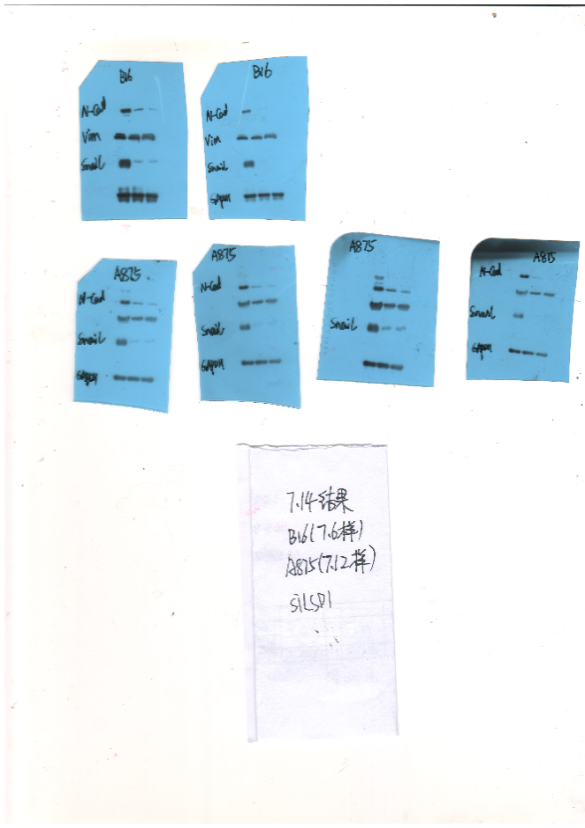


Fig 2C


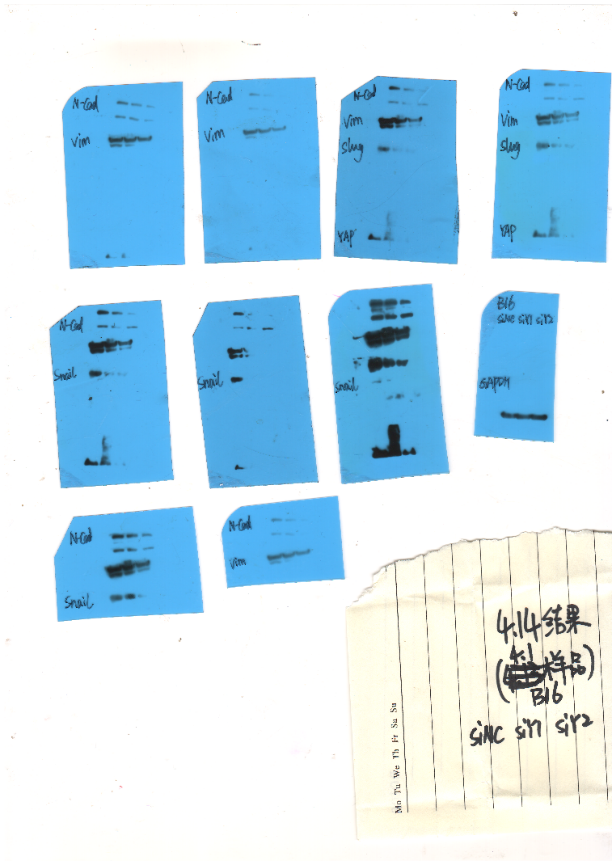

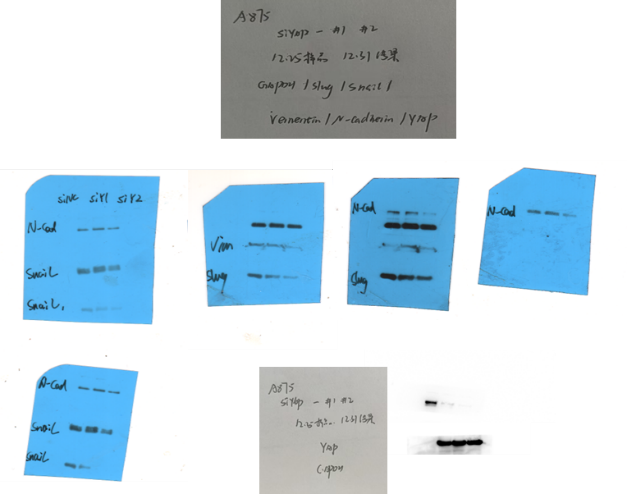


Fig 2D


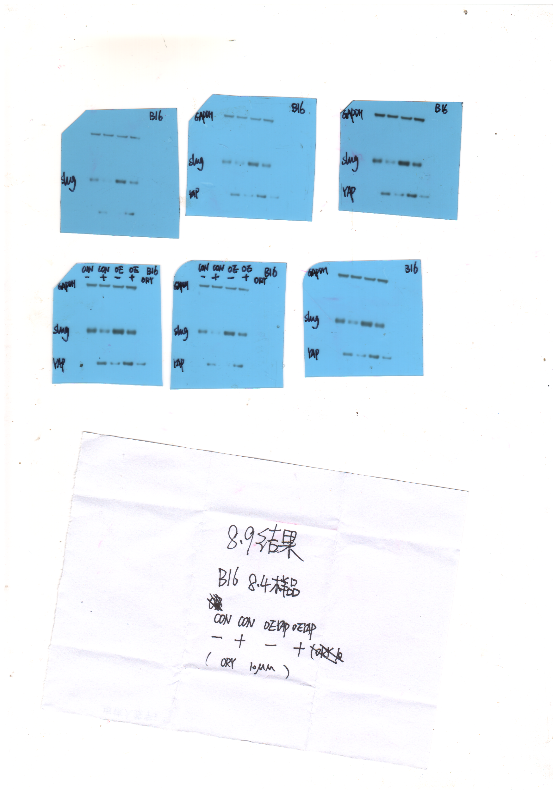

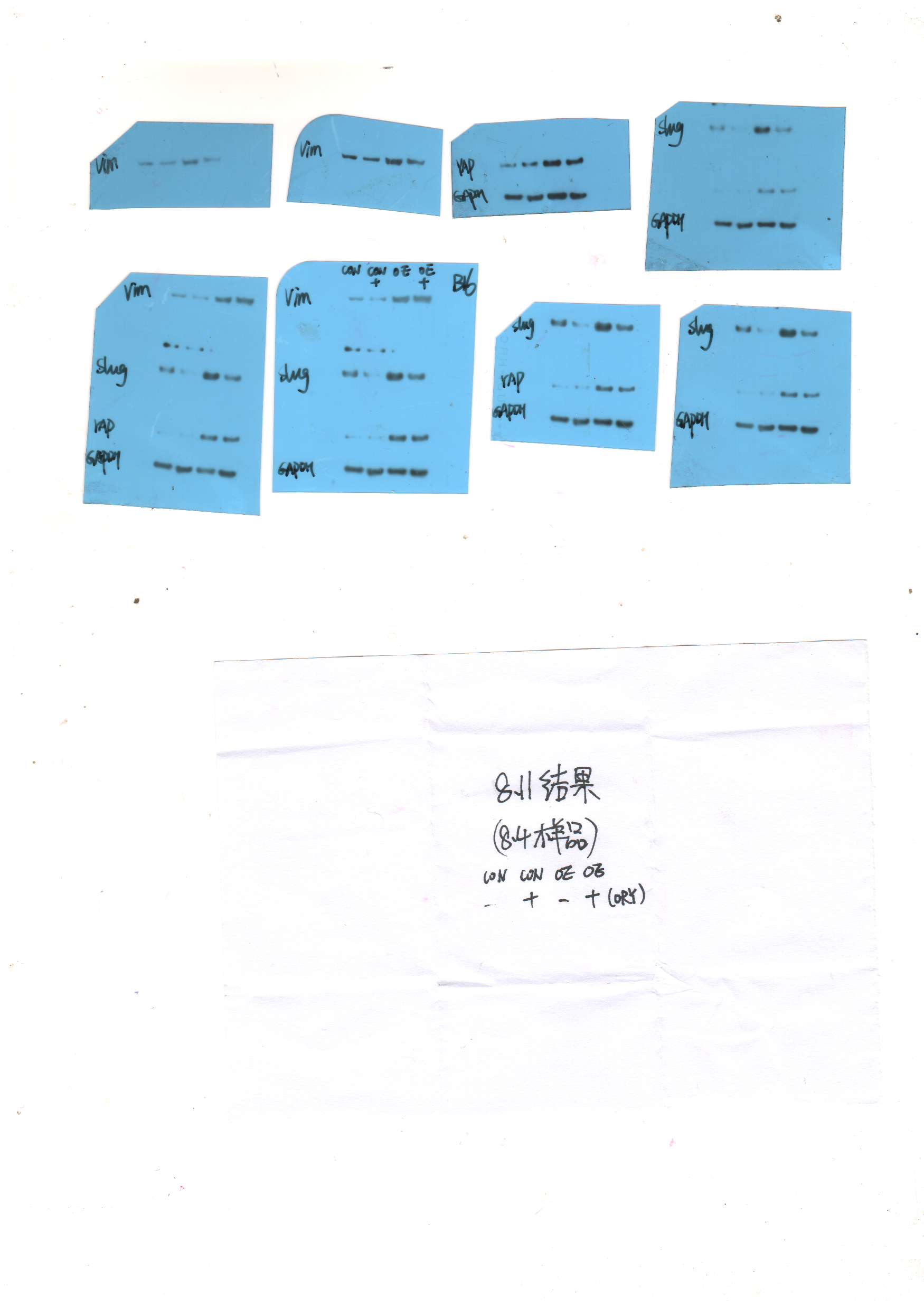

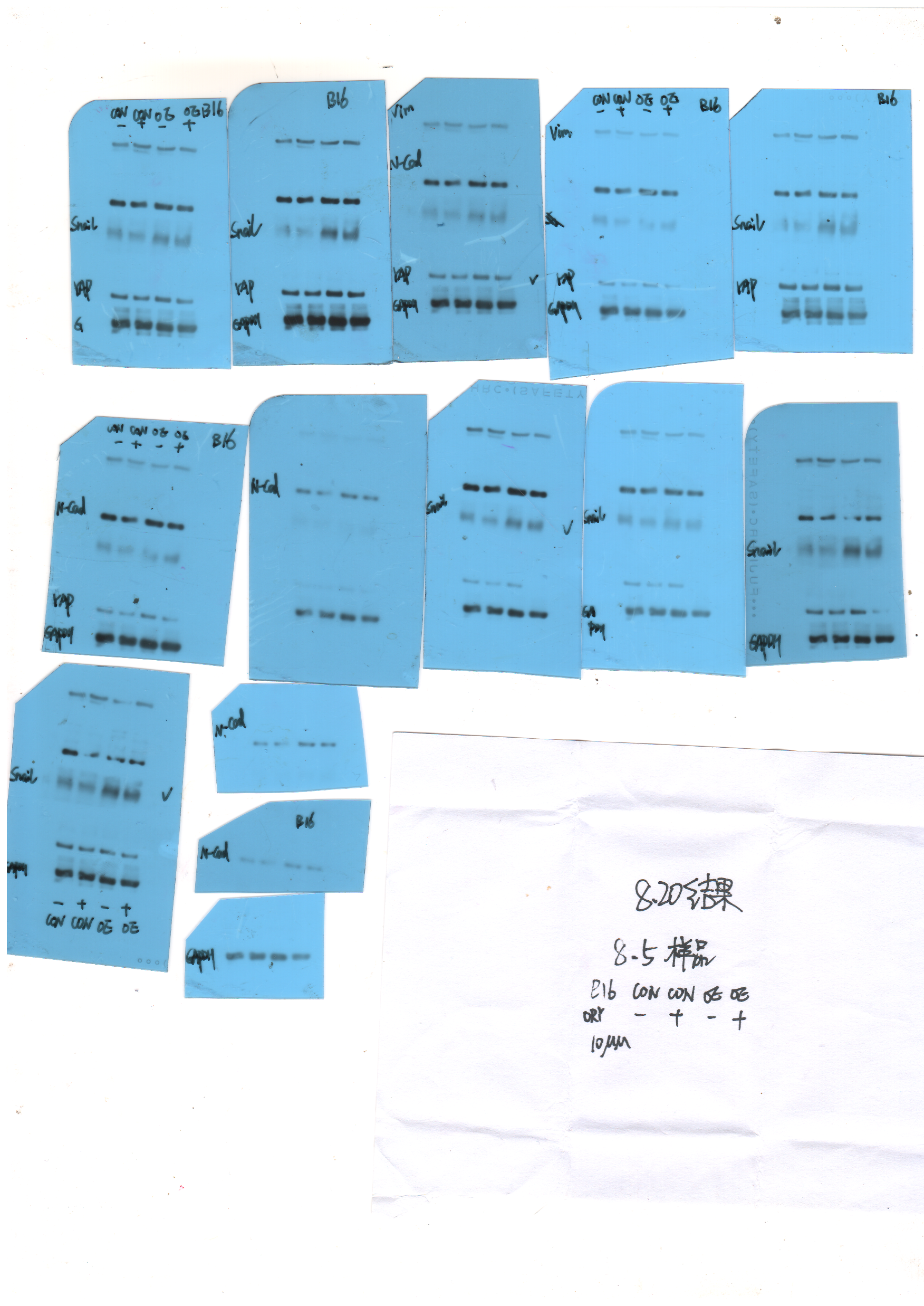

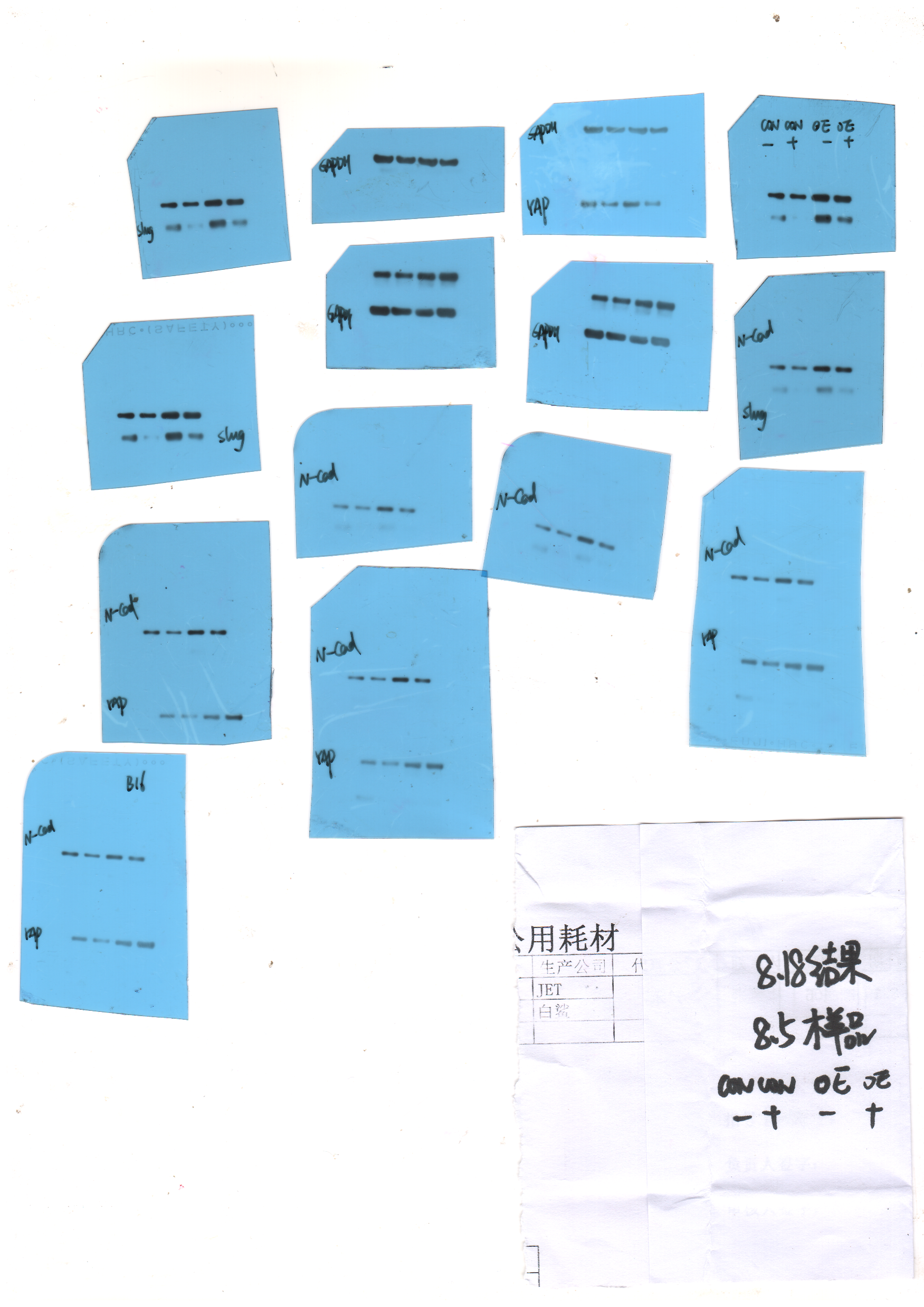

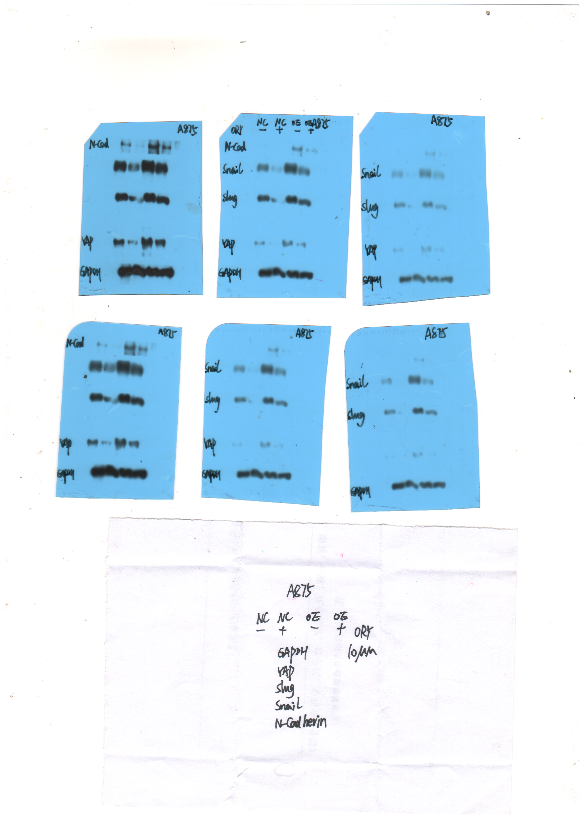

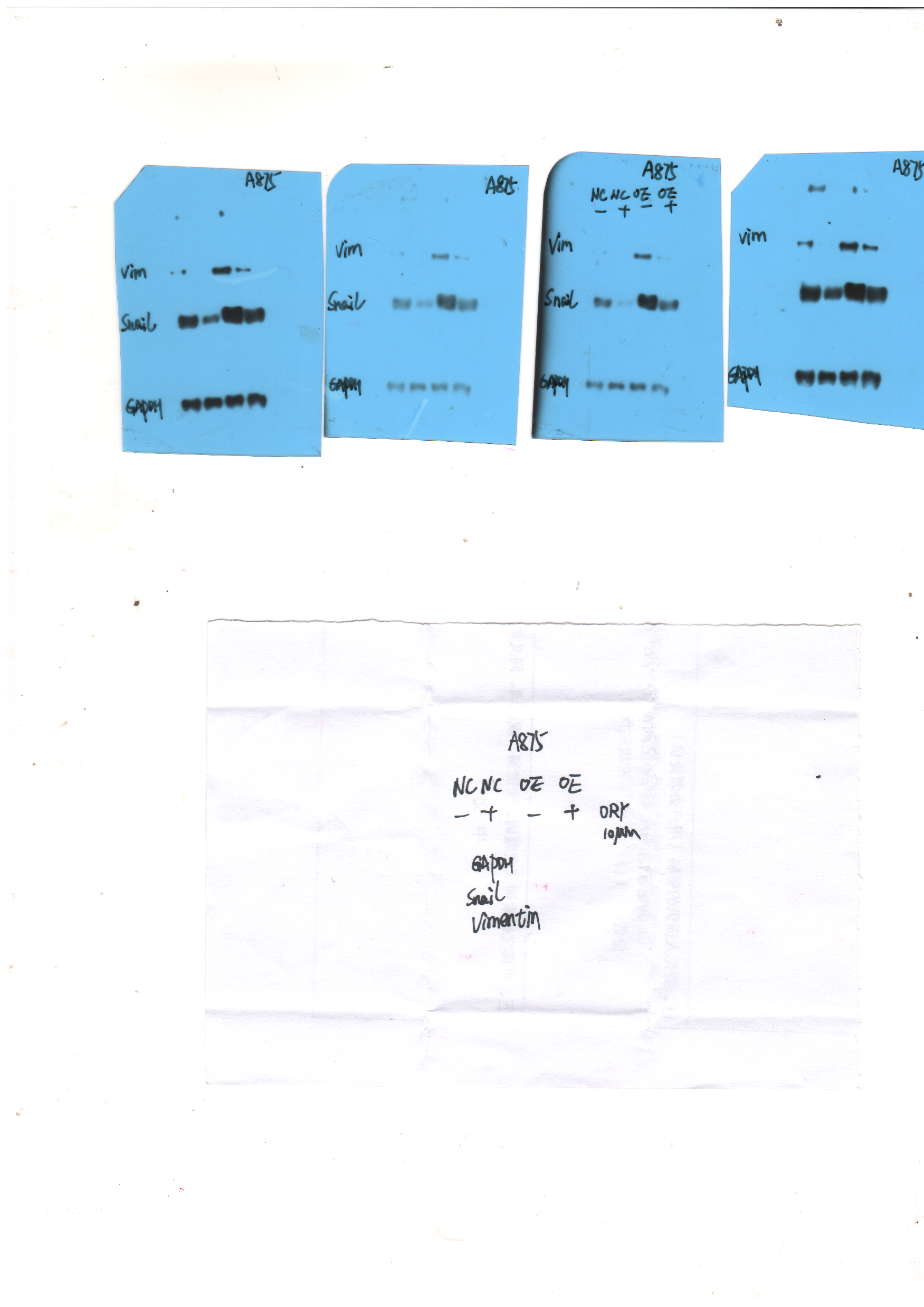


Fig 2I


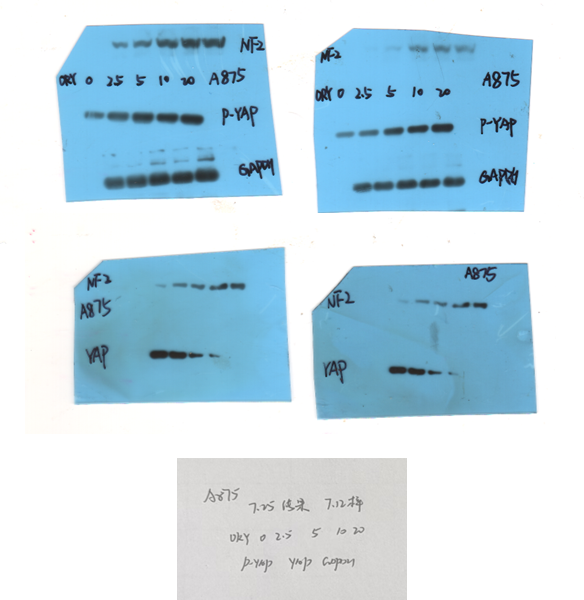


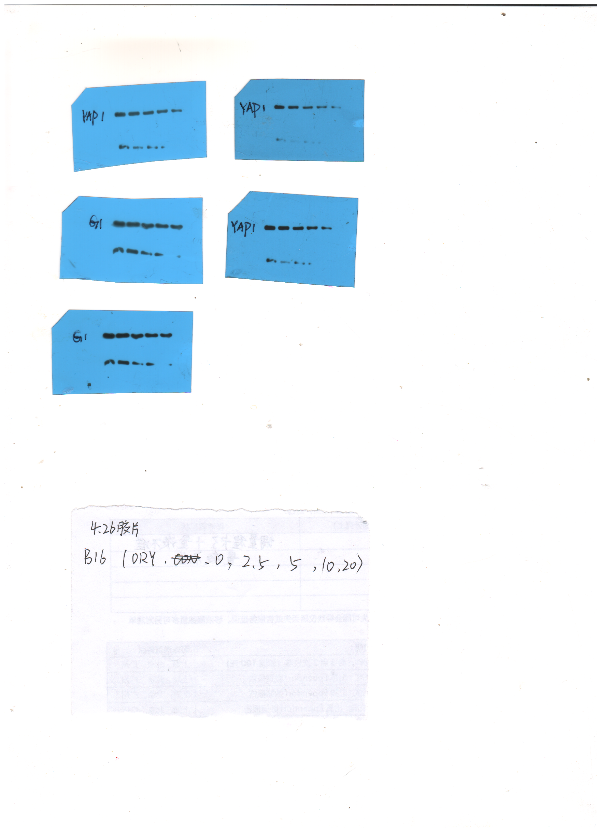

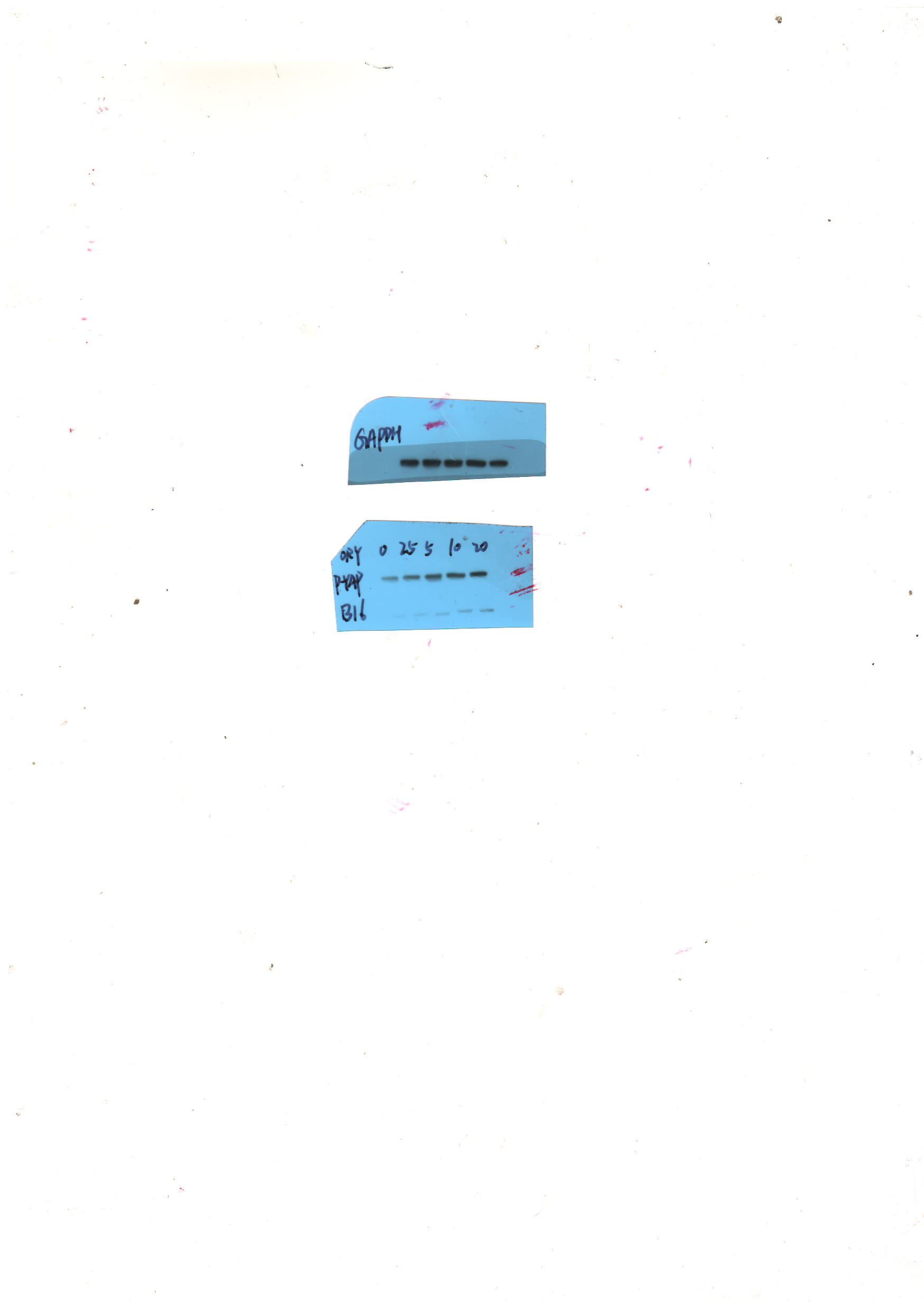


Fig 2J


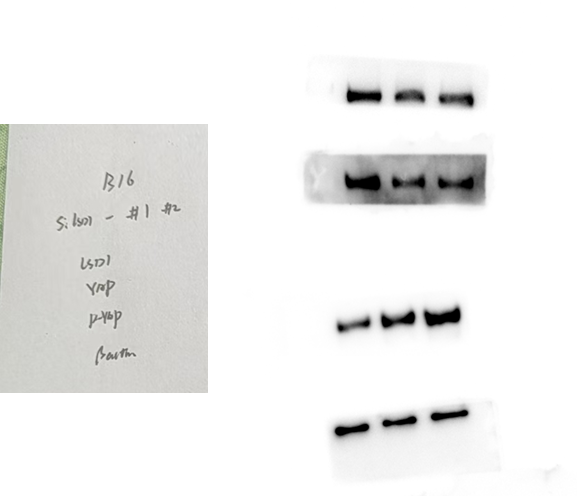

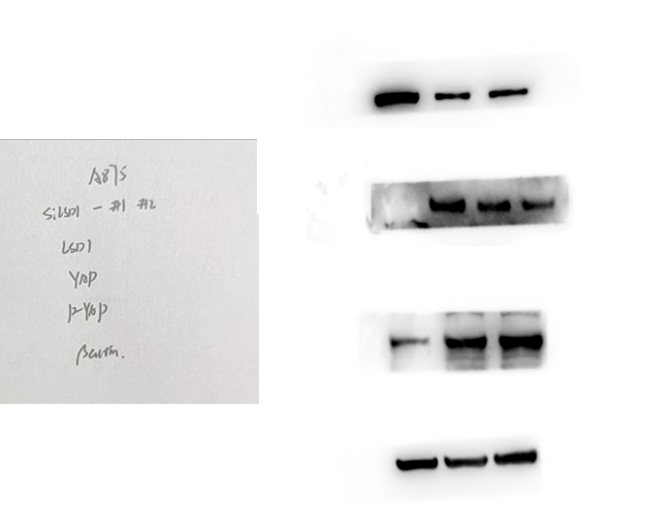


Fig 2K


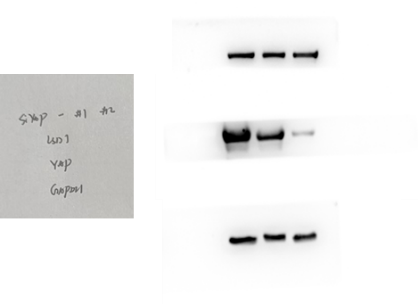

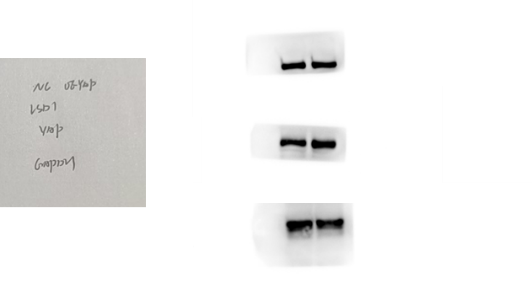


Fig 3C


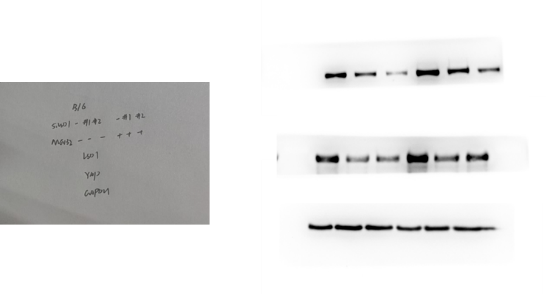

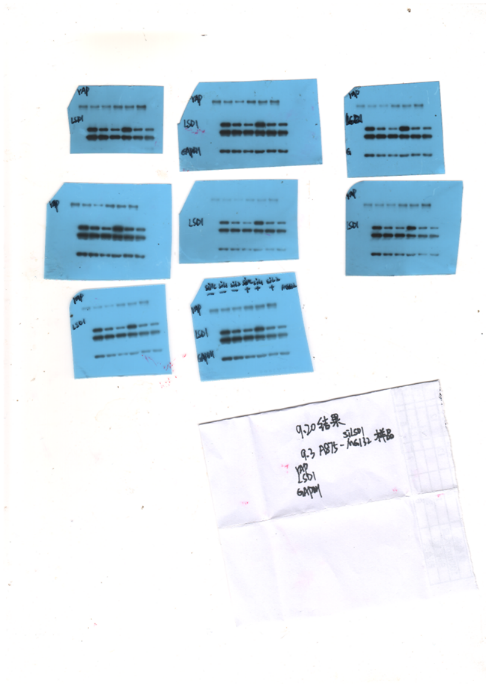


Fig 3D


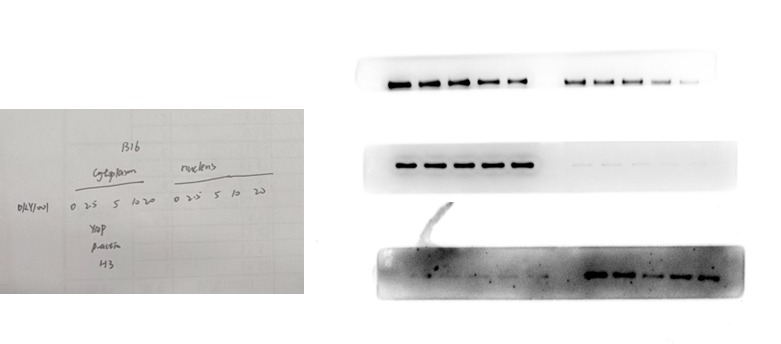


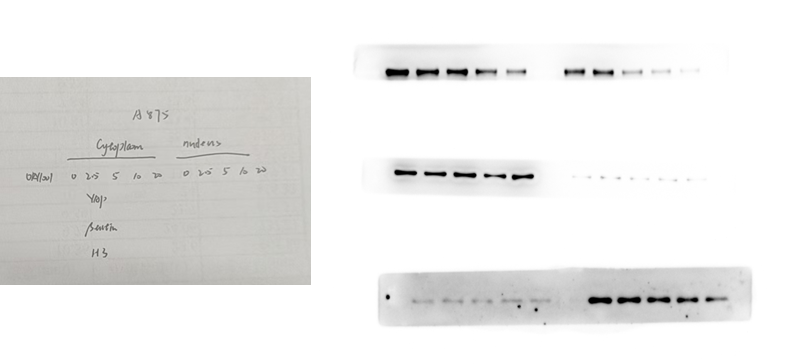


Fig 3E


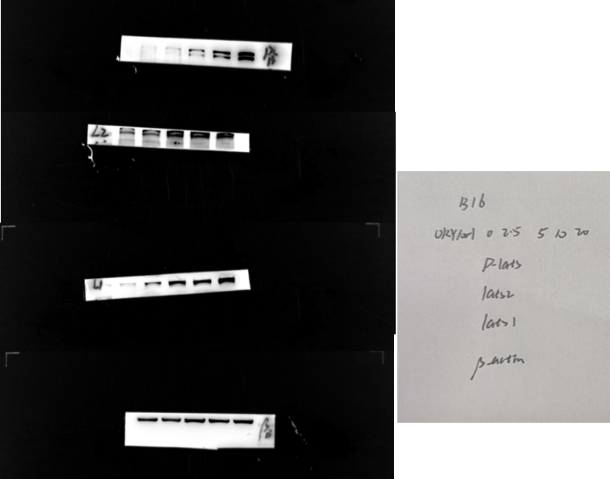

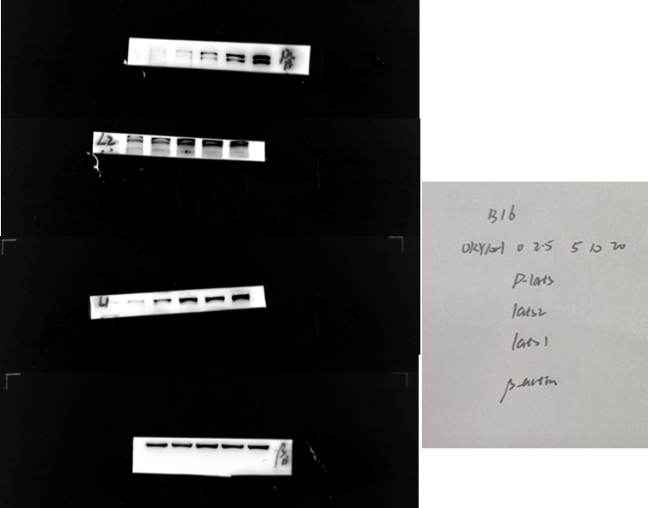


Fig 3F


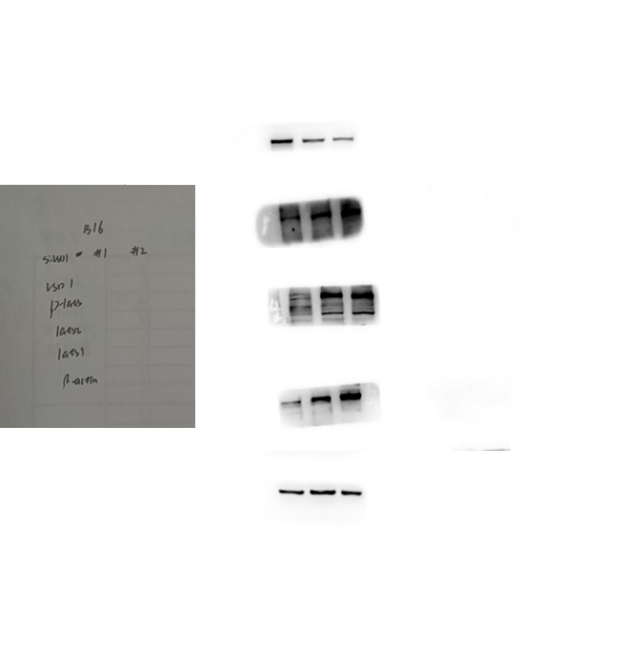

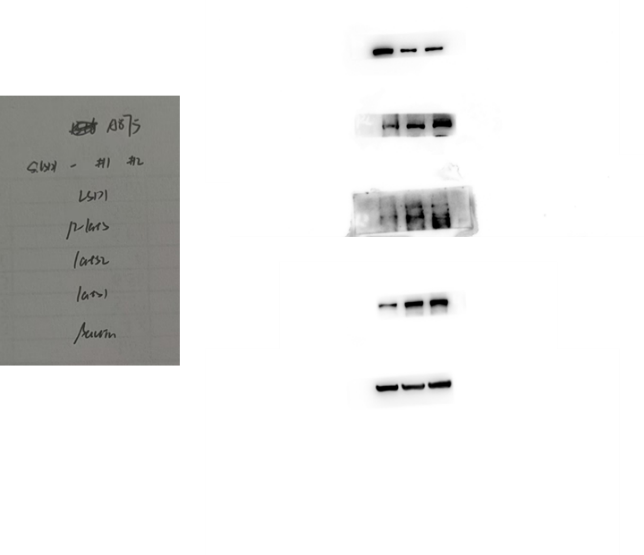


Fig 3G


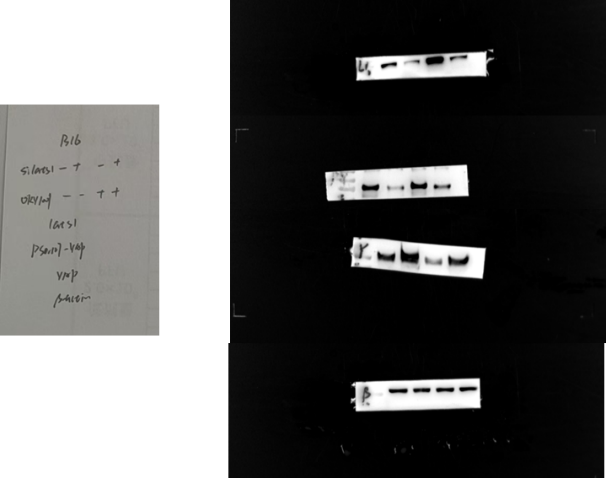

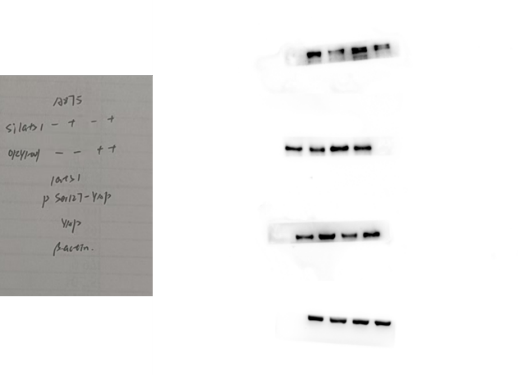


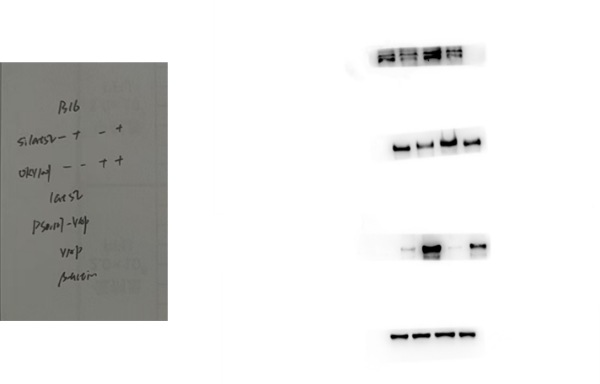

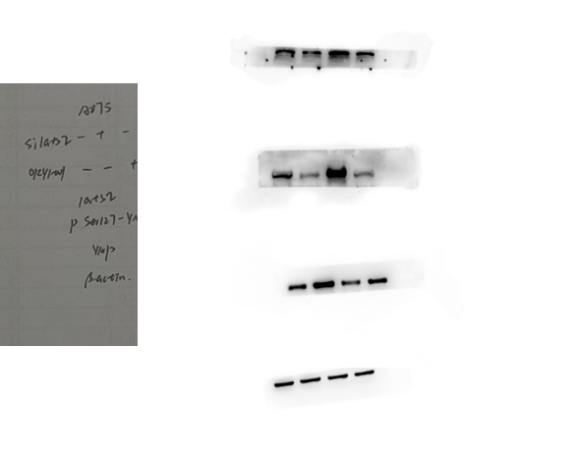


Fig 4A


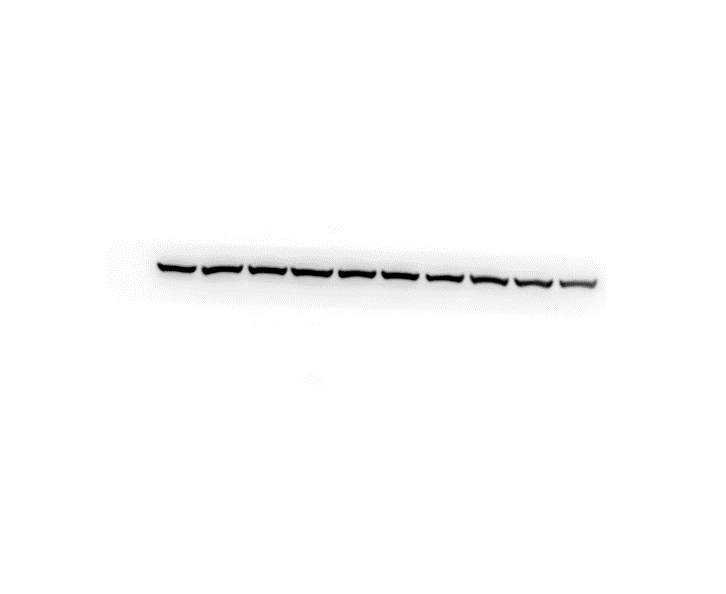

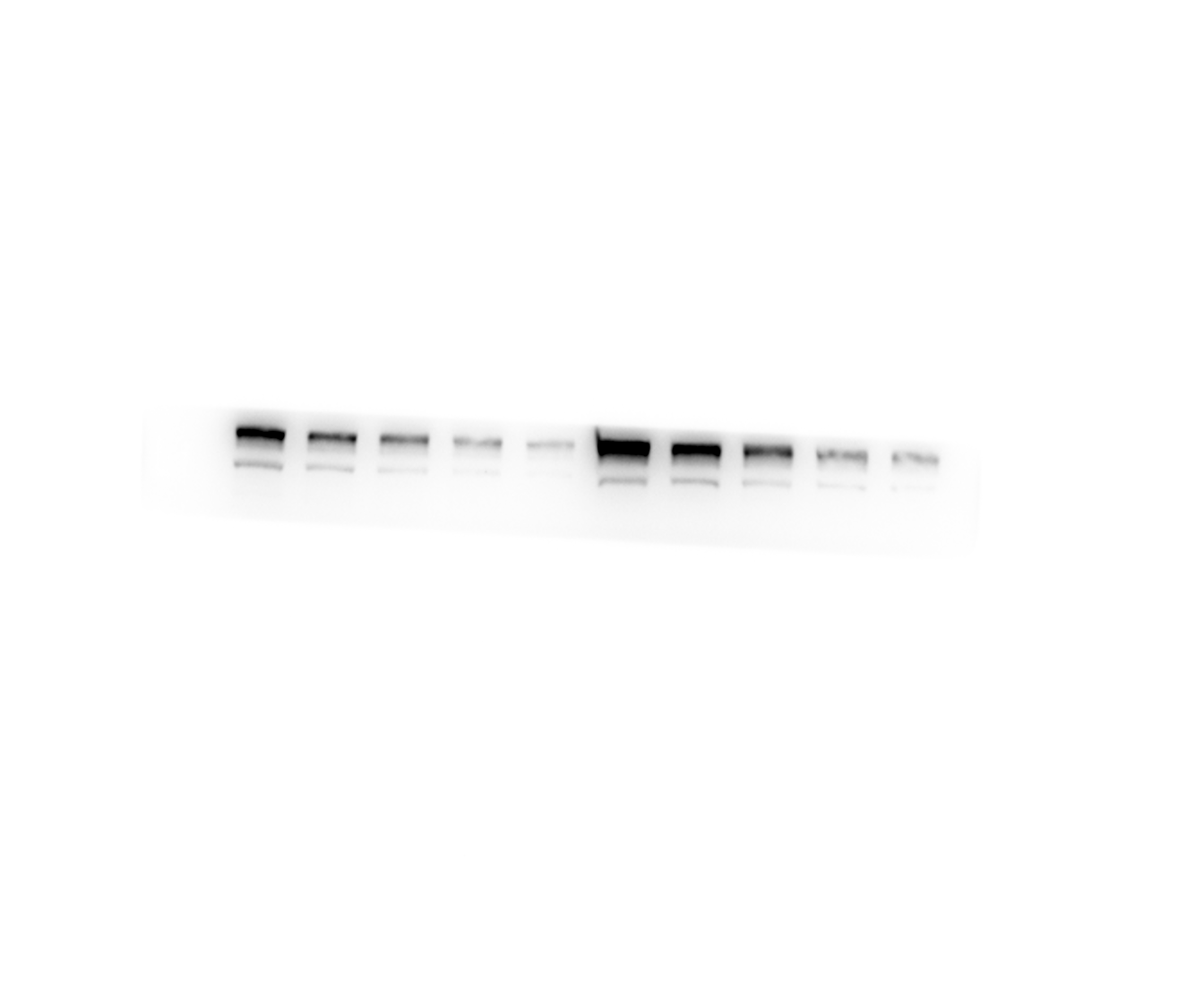

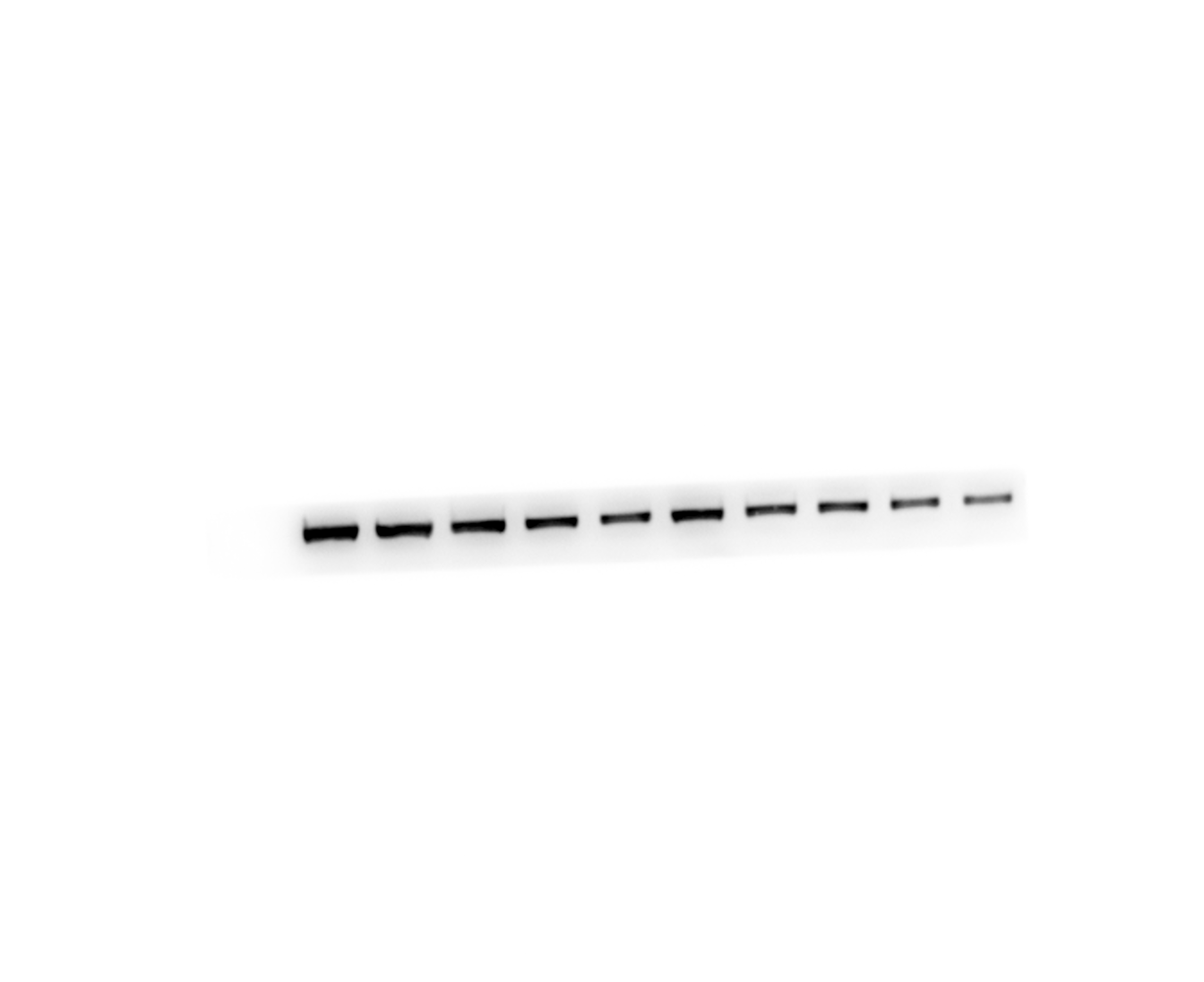

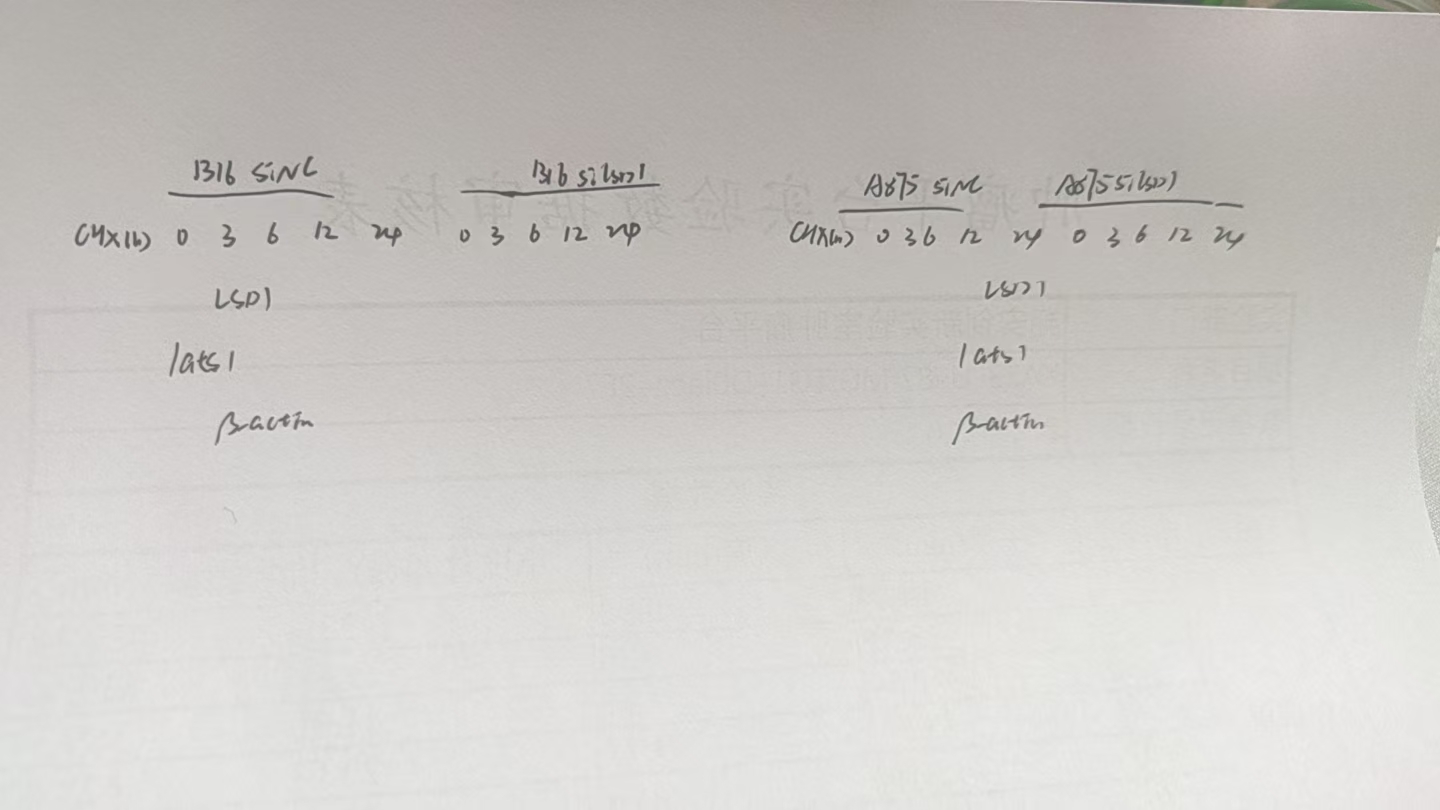


Fig 4B


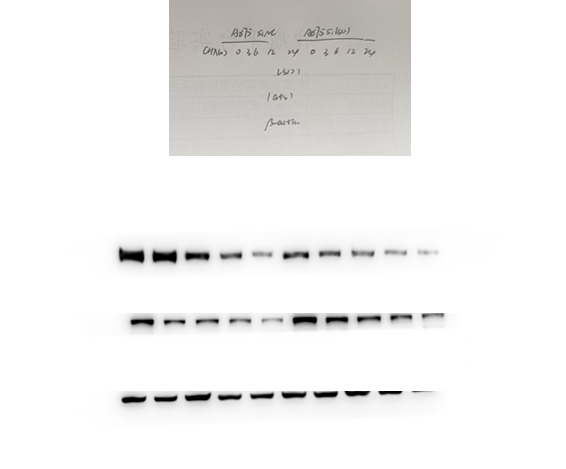


Fig 5A


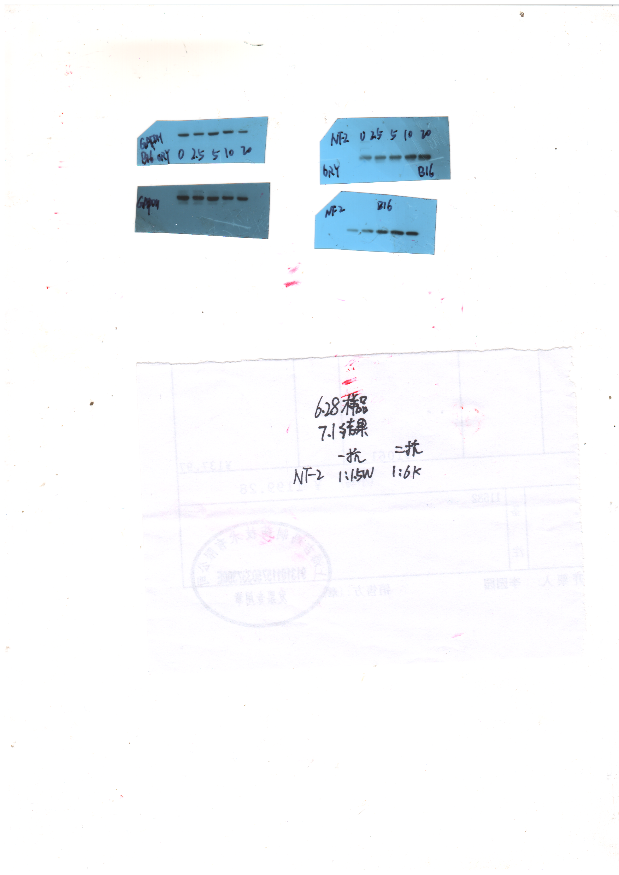

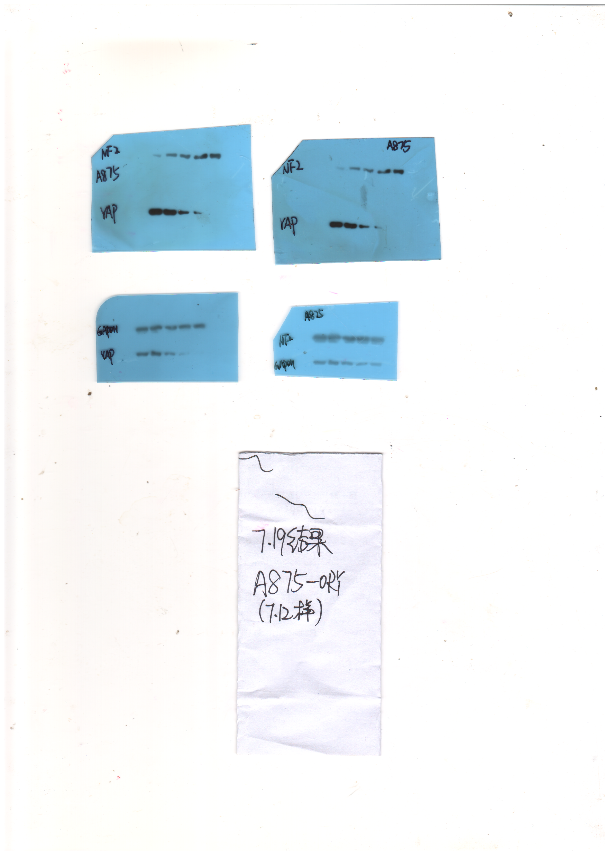


Fig 5B


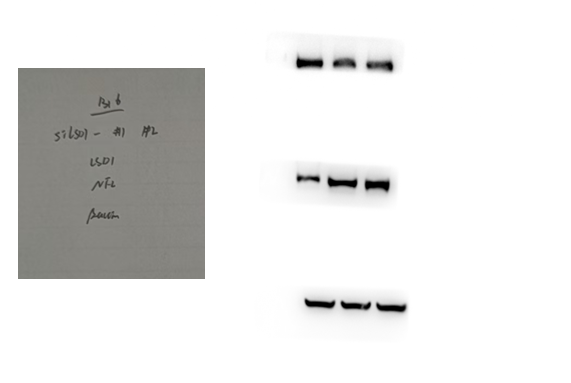

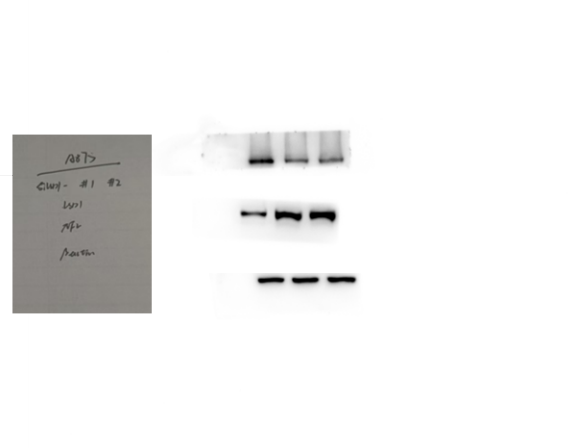


Fig 5C


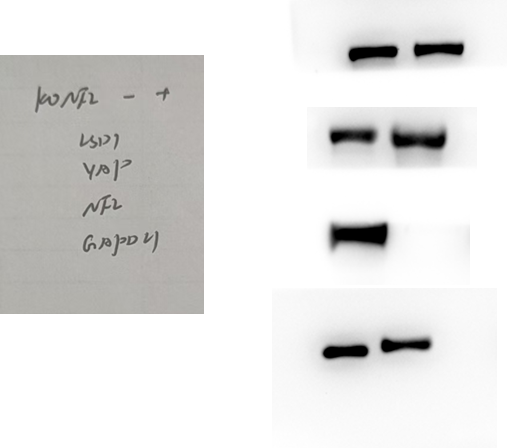


Fig 5D


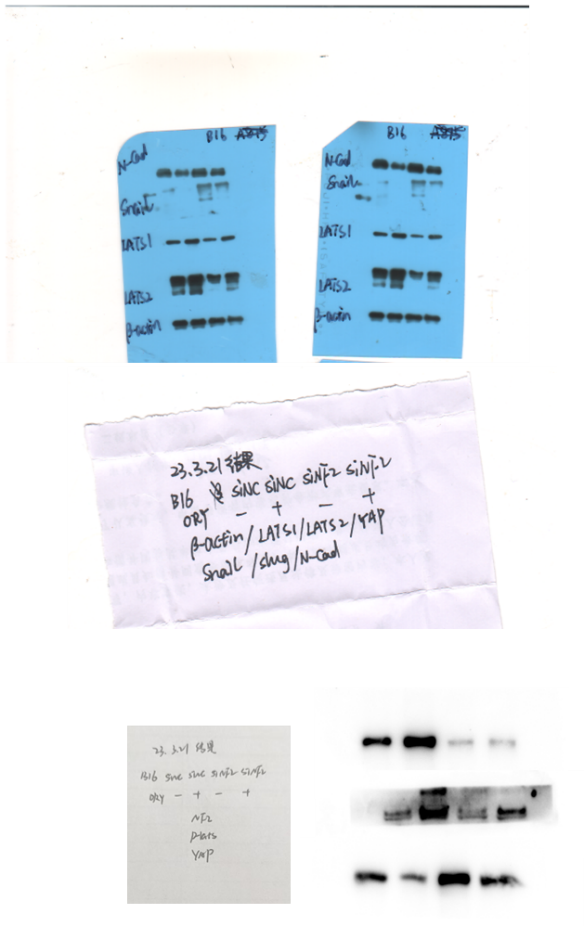

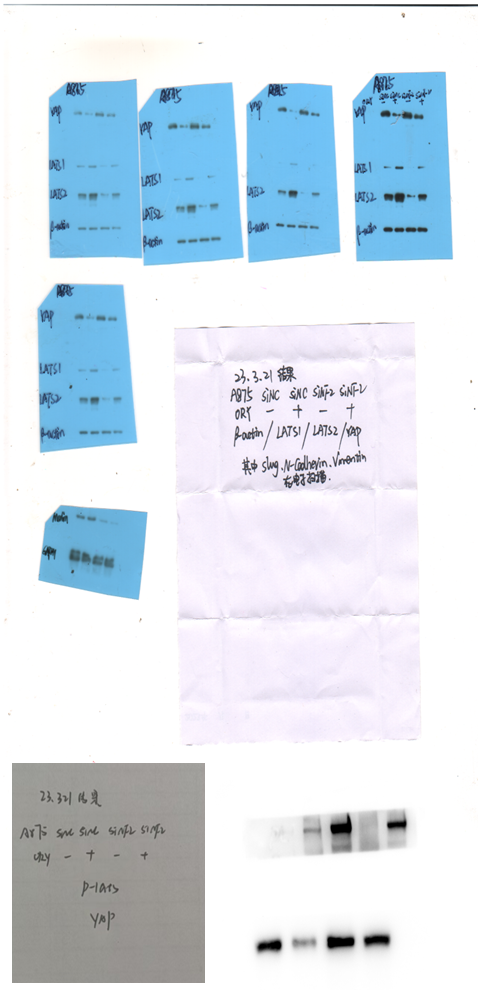


Fig 5E


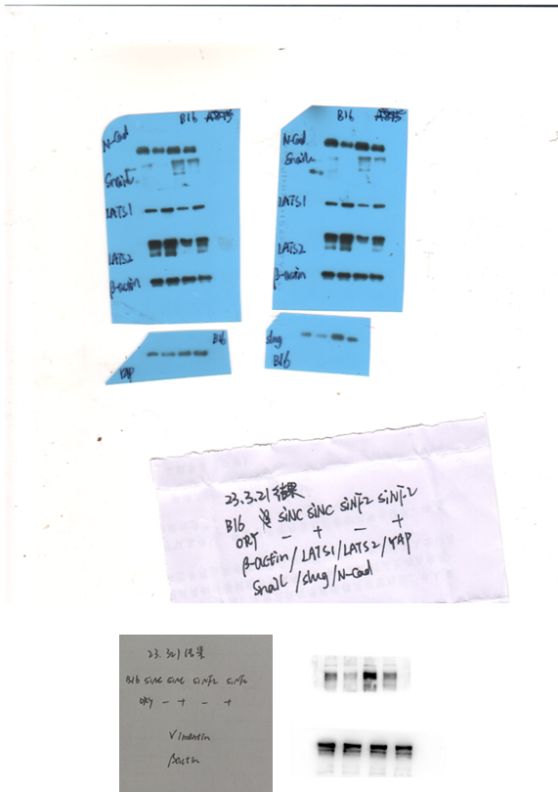

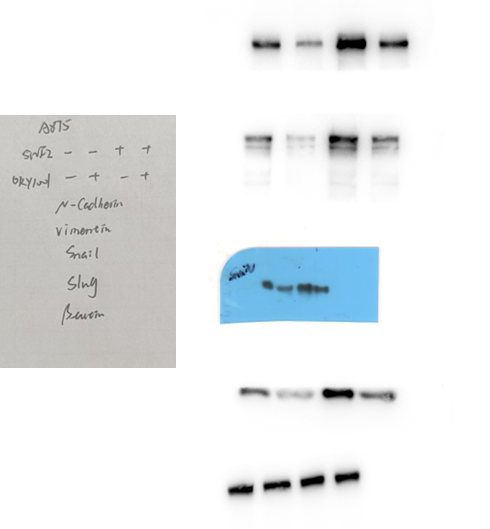


Fig 6A


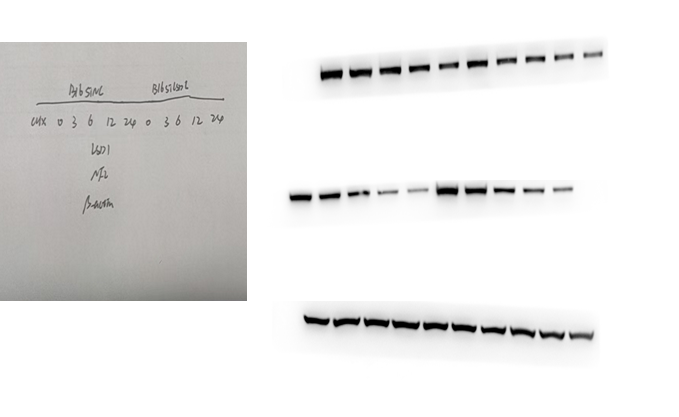


Fig 6B


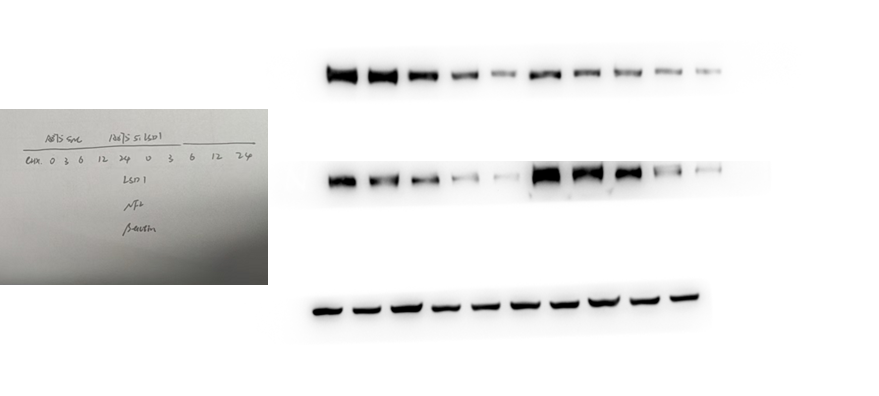

Supplement: Supplementary file 19 — original data [file 41419_2026_8872_MOESM19_ESM.docx]
